# Experimental Study on the Transfer of Polychlorinated Biphenyls (PCBs) and Polychlorinated Dibenzo-*p*-dioxins and Dibenzofurans (PCDD/Fs) into Milk of High-Yielding Cows during Negative and Positive Energy Balance

**DOI:** 10.1021/acs.jafc.3c02776

**Published:** 2023-08-31

**Authors:** Torsten Krause, Julika Lamp, Karin Knappstein, Hans-Georg Walte, Jan-Louis Moenning, Joachim Molkentin, Florian Ober, Andreas Susenbeth, Edwin Westreicher-Kristen, Karl-Heinz Schwind, Sven Dänicke, Peter Fürst, Hans Schenkel, Robert Pieper, Jorge Numata

**Affiliations:** † Department of Safety and Quality of Milk and Fish Products, Max Rubner-Institut (MRI), Hermann-Weigmann-Str. 1, 24103 Kiel, Germany; ‡ Department Safety in the Food Chain, German Federal Institute for Risk Assessment (BfR), Max-Dohrn-Straße 8-10, 10589 Berlin, Germany; § Institute of Animal Nutrition and Physiology, Kiel University (CAU), 24118 Kiel, Germany; ∥ Department of Quality and Safety of Meat, Max Rubner-Institut (MRI), E.-C.-Baumann-Str. 20, 95326 Kulmbach, Germany; ⊥ Institute of Animal Nutrition, German Federal Research Institute for Animal Health, Friedrich-Loeffler-Institut (FLI), Bundesallee 37, 38116 Braunschweig, Germany; # Institute of Food Chemistry, University of Münster, Corrensstrasse 45, 48149 Münster, Germany; ∇ Department of Animal Nutrition, University of Hohenheim, Emil-Wolff-Str. 10, 70599 Stuttgart, Germany

**Keywords:** food safety, feed safety, toxicokinetics, feed to food transfer, carryover

## Abstract

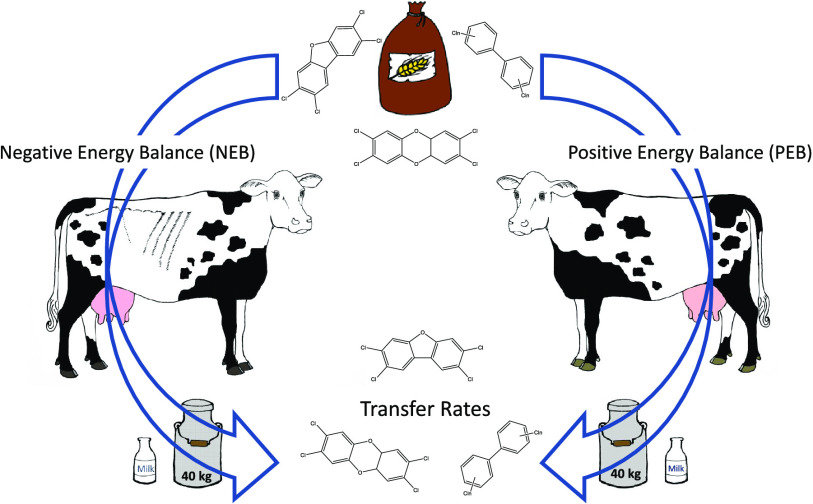

Dioxin-like polychlorinated biphenyls (dl-PCBs) as well as polychlorinated dibenzo-*p*-dioxins (PCDDs) and dibenzofurans (PCDFs) are a major concern for food safety, especially in fat-containing foods of animal origin, such as milk. Due to the lipophilic character of PCDD/Fs and PCBs, it is of special interest to explore whether the metabolic state of high-yielding cows influences the transfer rates into milk. Five German Holstein cows were orally exposed to a mixture of 17 PCDD/Fs, 12 dl-PCBs, and 6 non-dioxin-like PCBs (ndl-PCBs) for two dosing periods of 28 days each. The first period covered the negative energy balance (NEB) after calving, while the second period addressed the positive energy balance (PEB) in late lactation. Each dosing period was followed by a depuration period of around 100 days. During the NEB phase, the transfer rates of 14 PCDD/Fs and 7 dl-PCBs quantified were significantly (*p* ≤ 0.1) higher compared to the PEB phase, indicating an influence of the metabolic state on the transfer. Furthermore, the congener-specific transfer rates (0.3–39%) were in the range of the results from former studies. This indicates that the milk yield of the exposed cows is not the only determining factor for the transfer of these congeners into milk.

## Introduction

Polychlorinated biphenyls (PCBs) as well as polychlorinated dibenzo-*p*-dioxins (PCDDs) and dibenzofurans (PCDFs) are persistent organic pollutants (POPs). Due to their lipophilic and persistent nature, these substances are able to accumulate through the food chain and become an exposure source for consumers via fat-containing foods of animal origin, such as dairy products,^1^ eggs, meat, or fish.^2^ PCDDs and PCDFs are collectively and colloquially called “dioxins”. A subgroup of the PCB congeners with similar toxicity profile and molecular conformations are called dioxin-like PCBs (dl-PCBs); the rest of the congeners are non-dioxin-like PCBs (ndl-PCBs). PCB congeners are identified with a number under the Ballschmiter scheme.^3^

The mechanism of chronic toxicity is considered the same for PCDD/Fs and dl-PCBs, but their potency is congener-specific; thus, they are rated by the World Health Organization (WHO) according to their potency compared to the most toxic dioxin congener, 2,3,7,8-tetrachlorodibenzo-*p*-dioxin (TCDD), using toxic equivalency factors (TEFs). The WHO total toxic equivalency (WHO_2005_-TEQ) is calculated as a weighted sum using the currently established WHO_2005_-TEFs for seventeen PCDD/Fs and twelve dioxin-like PCBs (dl-PCBs).^4^ Concentrations in TEQ units are used to define action and maximum levels (ML) in feed and food safety frameworks, e.g., in the European Union.^5−7^ Furthermore, six non-dioxin-like PCBs (ndl-PCBs), which cover around 50% of total ndl-PCBs in feed and food, are used as indicator congeners for risk assessment and risk management.^8^

In the last decades, many feeding and field studies investigated the transfer of PCDD/Fs and PCBs along the feed-milk chain, as has been reviewed recently.^9,10^ Transfer studies with high-yielding cows^11,12^ are still rare, whereas even recent studies employed low (<15 kg milk/day)-^13^ to medium (<30 kg milk/day)^14−16^-yielding cows. Two open questions are whether the metabolic status of the cow has an influence on the transfer of contaminants and whether transfer parameters derived from older studies are still applicable to high-yielding dairy cow breeds.^9^

The balance between catabolic and anabolic processes has a direct impact on body fat reserves, which are the main compartment for the storage of lipophilic xenobiotics. This may be especially relevant for high-yielding dairy cows, which can exhibit a markedly negative energy balance (NEB) resulting from an imbalance between feed intake and energy loss via increasing milk production especially in the first weeks after calving.^17^ During NEB, a cow mobilizes body fat reserves meeting the energy requirement, including that for milk fat production, whereby stored lipophilic xenobiotics are also mobilized and secreted with milk while reducing the body fat compartment that stores such substances ingested with feed at the same time. This may effectively shift the direction of exchange of lipophilic compounds between blood and body fat^18^ so that more PCDD/Fs and PCBs move into the blood.^19^ During NEB, these processes could result in increased transfer rates (TRs) of lipophilic contaminants from feed to milk compared to later lactation, when the cow regains a positive energy balance (PEB).

Furthermore, intensive breeding programs over the last decades have resulted in significantly increased yields of milk and milk fat in specialized dairy breeds with up to 60 kg milk/day or more.^20^ Many older studies presenting congener-specific PCDD/F and PCB transfer parameters were conducted with cows with a lower yield, i.e., under 30 kg/day milk and under 1.2 kg/day milk fat.^13,15,21,22^ To date, only one study used high-yielding dairy cows with a milk production of about 40 kg/day and a milk fat yield of 1.5 kg/day.^11^ As milk fat is the main excretion route for many highly lipophilic substances like PCDD/Fs and PCBs in lactating animals, it becomes necessary to study transfer parameters using today’s high-yielding dairy cow breeds.

The aim of this study was to determine the congener-specific transfer of PCDD/Fs and PCBs in high-yielding dairy cattle considering the potential influence of the animals’ metabolic state. Therefore, a transfer study was conducted with nine high-yielding German Holstein cows. Four control cows were sampled over the entire experiment in order to account for the background exposure to PCDD/Fs and PCBs. Five experimental cows received an artificial mixture in capsule form containing all seventeen 2,3,7,8-substituted PCDD/Fs, twelve dl-PCBs, and six ndl-PCBs. To compensate for cow individual effects on the transfer and to give a better estimation of the influence of the animals’ metabolic state, the same cows were dosed twice: first during the NEB phase after calving and again during the PEB phase during late lactation for 28 days, respectively.

## Materials and Methods

### Ethics Approval Statement

All experimental procedures involving animals were approved by the Ministry of Energy, Agriculture, the Environment, Nature, and Digitalization of Schleswig-Holstein, Germany (reference number V241-37856/2017; 102-8/16).

### Experimental Design

The transfer study was carried out at the agricultural research station of the Max Rubner-Institut in Schaedtbek, Germany, from October 2016 to January 2018. Cows were kept in an outdoor climate freestall barn with slatted floor and deep straw bedding cubicles. From this herd, nine healthy and lactating cows, German Holstein breed, black and white within their second pregnancy were selected (Table 1). These cows were randomly allocated to an original experimental group of five and an original control group of four individuals. Around calving, cows were kept in deep straw calving boxes. After calving, both experimental and control cows were relocated to a separate section of the barn.

**Table 1:** Overview of Experimental Group and Control Group

group	cow ID	date of calving	date of death	
experimental	3425	03.11.2016	06.09.2017	euthanized
	3426	08.11.2016	06.09.2017	euthanized
	3438	05.02.2017	13.12.2017	euthanized
	3441	16.02.2017	19.07.2017	died
	3448	06.03.2017	10.01.2018	euthanized
control	3419	30.10.2016	13.09.2017	euthanized
	3420^a^	31.03.2017	10.01.2018	euthanized
	3432	26.02.2017	13.12.2017	euthanized
	3434	07.11.2016	18.05.2017	died

a Replacement for cow 3434.

All animals were fed for ad libitum feed intake with a mineralized mixed ration of grass and maize silage. They received additional concentrate feed from a computer-controlled feeding station according to their individual milk yield. The total forage intake of the group was determined by weighing the daily amount of feed offered and feed residues on the following day, allowing an estimation of the individual daily forage intake. At the end of the study, the cows were euthanized under general anesthesia and the dissection was performed at the Department of Pathology of the University of Veterinary Medicine in Hannover, Germany. The results of the dissection will be part of another publication. During the experiment, two animals (control cow 3434 and experimental cow 3441) died untimely as a result of a chronic metabolic disease (see the Clinical Findings in Cows 3434 and 3441 section). Both were dissected at the responsible rendering plant (Jagel, Germany) by a specialized veterinarian. Cow 3434 was replaced by cow 3420.

### PCDD/F and PCB Dosing Regimen

The dosing of the cows was carried out during lactation in two phases, each lasting 28 consecutive days. The first phase started on day 1 after calving. The second phase was started when the cows were in a positive energy balance after around 6 months of lactation (variable for each cow, see the Evaluation of Cow Energy Balance section). To ensure an exact daily dosage, water-soluble capsules (Science Services GmbH, Munich, Germany) containing ground concentrate feed fortified with a mixture including all seventeen 2,3,7,8-substituted PCDD/Fs, all twelve dl-PCBs, and six ndl-PCBs (see the Chemicals section) with the concentration adjusted with picograde n-hexane (LGC Standards, Teddington, England) were prepared individually for each cow prior to the respective dosing phase. The concentrations of PCDD/Fs and PCBs were analyzed by gas chromatography/high-resolution mass spectrometry (GC-HRMS) in each batch to verify the individual dose (WHO_2005_-TEQ: Table 2; congener-specific dosing: Tables S1 and S2). The capsules were sealed with 1% citric acid solution and administered orally with a cattle bolus gun every morning of the dosing periods after milking.

**Table 2:** Details on WHO_2005_-TEQ Dosing Regimen Per Day and Per kg Body Weight (BW; See Table 3)

	first dosing (day 1–28 p.p.)				second dosing (around day 179–206 p.p.)			
cow ID number	∑PCDD/Fs [ng/day]	∑dl-PCBs [ng/day]	∑PCDD/F+ dl-PCB [ng/day]	∑ndl-PCBs [μg/day]	∑PCDD/Fs [ng/day]	∑dl-PCBs [ng/day]	∑PCDD/F+ dl-PCB [ng/day]	∑ndl-PCBs [μg/day]
3425	118.86	41.65	160.51	39.73	120.71	41.65	162.36	44.91
3426	118.86	41.65	160.51	39.73	120.71	41.65	162.36	44.91
3438	138.92	51.19	190.11	50.69	143.10	51.19	194.29	49.72
3441	138.92	51.19	190.11	50.69	cow died			
3448	153.59	55.67	209.26	57.72	153.59	55.67	209.26	57.72

first dosing (day 1–28 p.p.)					second dosing (around day 179–206 p.p.)			
cow ID number	∑PCDD/Fs [pg/kg BW]	∑dl-PCBs [pg/kg BW]	∑PCDD/F+ dl-PCB [pg/kg BW]	∑ndl-PCBs [ng/kg BW]	∑PCDD/Fs [pg/kg BW]	∑dl-PCBs [pg/kg BW]	∑PCDD/F+ dl-PCB [pg/kg BW]	∑ndl-PCBs [ng/kg BW]
3425	184.61	64.70	249.31	61.71	190.24	66.37	256.61	70.77
3426	181.81	63.72	245.53	60.77	174.61	60.92	235.53	64.96
3438	206.24	76.00	282.25	75.26	203.75	74.59	278.33	70.80
3441	216.33	86.69	303.02	89.88	cow died			
3448	228.49	82.82	311.31	85.87	214.14	77.62	291.76	80.47

### Clinical Examination

All animals were examined regularly by a veterinarian to document their general health and clinical appearance including behavior, feed and water intake as well as urination and defecation. Blood β-hydroxybutyrate values were measured regularly per rapid test (Nova vet, Nova Biomedical Taiwan Corporation, New Taipei City, Taiwan). The individual daily milk yield was documented. Animals showing signs of illness were examined clinically and treated if necessary. Body weight (BW) was measured regularly using a continuous weighing scale at the exit of the milking parlor.

### Sampling and Sample Preparation

During the experiment, milk, feed, and feces samples were taken from all animals according to the sampling scheme (Figure S1), with higher frequency at the beginning of the dosing and depuration periods. Control cows were sampled less frequently for background residue analysis.

Milk samples were taken for analysis of PCDD/Fs and PCBs, milk composition, and fatty acids according to the scheme presented in Figure S1. The cows were milked in a tandem milking parlor (GEA Farm Technologies, Bönen, Germany) twice a day at 7 am and 5 pm, and milk yields were recorded individually per cow. The milk of each cow was collected in a separate bucket and sampled after thorough mixing. Every milk sample for PCDD/F and PCB analysis consisted of morning milk and the evening milk of the previous day, each collected in a separate glass jar. The samples were stored at 4 °C until further processing within 7 days. Every week, four additional milkings were sampled for analysis of milk composition (100 mL) and analysis of fatty acid composition (100 mL) over three consecutive days, starting with evening milk. Feces (around 400 g each) were collected rectally from all cows according to the scheme in Figure S1. The sampling was always conducted at the same time of the day. All samples were stored at −20 °C until analysis.

Feed, premixed grass, and maize silage, as well as concentrate feed were sampled for PCDD/F and PCB analysis every month and stored at −20 °C.

#### Chemicals

Isotope-labeled ^13^C calibration (PCDD/Fs: EDF-9999, 1/10 concentration; dl-PCBs: EC-5380; ndl-PCBs: EC-5385), extraction (PCDD/Fs: EDF-4139; dl-PCBs: EC-5372; ndl-PCBs: EC-5379), cleanup (PCDD/Fs: EDF-6999; PCBs: EC-5370), and internal injection (PCDD/Fs: EDF-5999, PCBs: EC-5371) standards for GC-HRMS analysis as well as most native 2,3,7,8-chlorinated PCDD/Fs certified standards for the dosing regimen were from Cambridge Isotopes Laboratories, Inc. (Andover, MA). Native 2,3,7,8-TCDD, OCDF, dl-PCBs, and ndl-PCBs certified standards for the dosing regimen were from LGC Dr. Ehrenstorfer (Augsburg, Germany). The full list of all native congeners dosed and analyzed can be found in Tables S1 and S2.

Acetone, acetonitrile, toluene, *n*-hexane, dichloromethane, n-nonane, and petroleum benzine were of Picograde purity (LGC Standards, Teddington, England). Ethanol was of SupraSolv, ammonium sulfate of Emsure, and sodium sulfate of Emprove purity (Merck, Darmstadt, Germany). Glass filter (grade 595) was from Whatman plc (Maidstone, England). Samples for residue analysis were stored in certified glass jars (Thermo Fisher Scientific, Waltham, MA) according to U.S. Environmental Protection Agency “Specification and Guidance of Contaminant-Free Sample Containers’’.^23^

#### Analysis of Milk Composition

Milk samples collected for milk composition analysis were individually analyzed for fat, total protein, lactose, dry mass, somatic cell count, and urea using Fourier transform infrared spectroscopy at an official milk control laboratory (Landeskontrollverband Schleswig-Holstein, Kiel, Germany) twice a week. Furthermore, the pooled milk samples for PCDD/F and PCB analysis were analyzed for fat, total protein, and dry mass in-house with a MilkoScan 50 (type 71600, Foss Electric, Denmark).

#### Sample Extraction for PCDD/F and PCB Analysis

Milk samples, previously kept chilled, were heated to 40 °C in a water bath to allow homogenization of fat. Evening and morning milk were blended into a pooled sample per cow according to the respective milk yields in kilogram. Pooled milk was chilled to 4 °C and centrifuged at 3000 rpm for 40 min to separate the cream from milk. The cream was stored at −20 °C until PCDDF/s and PCBs were extracted by a modified Soxhlet extraction.^24,25^ In short, the cream was freeze-dried, blended with celite 545 (Merck, Darmstadt, Germany) and fat was extracted with petroleum benzine in a Twisselmann aperture for at least 6 h. The extract was concentrated with a rotary evaporator and the remaining solvent was removed during storage at 40 °C overnight. The remaining fat was aliquoted into 5 g samples and stored at −20 °C in glass vials.

Feces samples were weighed into 1 L glass bottles and subsequently lyophilized for at least 6 days to dryness. 10 g of the dried sample was spiked with extraction standards and extracted with an *n*-hexane/acetone mixture (ratio 2:1)^19,26^ in a Twisselmann-apparatus for at least 8 h. The extract was dried over sodium sulfate, filtered through a glass filter, and concentrated to about 1 mL with a rotary evaporator. The remaining solvent was removed during storage at 40 °C overnight. The fecal crude fat content, also known as ether extract, was determined gravimetrically. However, ether extract values for cow feces vary due to analytical methods^27,28^ and feeding regimes.^29,30^

For feed analysis, around 300 g of mixed grass and maize silage or 600 g of concentrate feed were weighed into 1 L glass bottles and subsequently lyophilized for at least 48 h to dryness. The extraction is based on the DIN EN 16215:2012-07 method. In short, the dried sample material was ground using a cutting mill (Retsch, Haan, Germany) and 10 g of each sample was spiked with extraction standards. Afterward, the samples were extracted in a Twisselmann-apparatus, first with toluene for at least 4 h and second with a toluene/ethanol mixture (ratio 9:1) for at least 8 h. The extracts were dried over sodium sulfate, filtered through a glass filter, and concentrated to about 1 mL with a rotary evaporator. The remaining solvent was removed during storage at 40 °C overnight.^31^

#### Sample Cleanup for PCDD/F and PCB Analysis

After thawing the fat extracts from milk, feces, and feed, they were dissolved in 2 mL of toluene and 3 mL of *n*-hexane and spiked with the cleanup standards for PCDD/F and PCB analysis. Additionally, the milk fat sample was fortified with the respective extraction standards. These solutions were transferred to a sample loop of a DexTech (LCTech GmbH, Obertaufkirchen, Germany) by rinsing the sample vials thrice with 3 mL of *n*-hexane. The DexTech consists of four columns for sample cleanup and separation of PCDD/Fs and PCBs in three different fat-free fractions applying different eluents.^32^ The first fraction contains the mono-ortho dl-PCBs and ndl-PCBs, the second fraction contains the non-ortho dl-PCBs, and the third fraction contains the PCDD/Fs.

The first fraction of the grass and feces samples was further purified by solid phase extraction (Chromabond C18 ec, Macherey-Nagel, Düren, Germany) using acetonitrile as eluent.

#### GC-HRMS Analysis of PCDD/Fs and PCBs

The solvent of the three fractions from the previous sample cleanup was concentrated first by rotary evaporations and subsequently, adding n-nonane as a keeper, under a gentle stream of nitrogen (99.999%, Alphagaz 1, Air Liquide, Paris, France) to a final sample size of 10 μL for fraction I and II containing the PCBs, and 5 μL for fraction III containing PCDD/Fs. Depending on the fractions and analytes, the samples were fortified with an adequate internal standard spiking solution prior to injection into the GC-HRMS.

The injection volume was 1 μL for PCBs in fractions I and II, and 2 μL for PCDD/Fs in fraction III. A splitless injection was performed in a 5890 series II (Hewlett-Packard, Palo Alto, CA) GC with an injector set at 280 °C. A ZB-SemiVolatiles (60 m, 0.25 mm ID, 0.25 μm film thickness, Phenomenex, Torrance, CA) with a siltek deactivated retention gap (5 m, 0.25 mm ID, Restek, Bellefonte, PA) was applied for chromatographic separation. The oven program was 120 °C, held for 3 min, 19 °C/min to 210 °C, 2 °C/min to 275 °C held for 7 min, 20 °C/min to 300 °C held for 11.5 min for PCDDFs and 120 °C held for 2 min, 20 °C/min to 180 °C, 1.25 °C/min to 255 °C, 40 °C/min to 300 °C held for 3.5 min for PCBs.

The temperature of the transferline was set at 280 °C, while the ion source of the MAT 95 (Finnigan MAT, Bremen, Germany) sectorfield MS was set at 250 °C. Analytes were ionized at 45 V and 0.5 A, and ions were detected in positive mode. For PCDD/F analysis, the resolution of the HRMS was set to 10,000, and for PCB analysis, it was set to at least 8000. Dioxins and PCBs were measured in multiple ion detection (MID) mode using FC-43 (MasCom GmbH, Bremen, Germany) as reference gas for lock and calibration peaks. The MID analysis is based on EPA Method 1613B^33^ and 1668C.^34^ In short, for every congener, four masses were recorded, including two for the isotope-labeled congeners in the extraction standard. Exceptions were 1,2,3,7,8,9-HxCDD and OCDF. The first is quantified using the average response of the two other isotope-labeled 2,3,7,8-HxCDD congeners from the extraction standard and the latter is quantified against the isotope-labeled OCDD. Chromatograms were analyzed using the Xcalibur software suite (2.0) including Quandesk (2.0 SP4).

Calibration and quantification were performed using CIL-EDF-9999-A for PCDD/Fs, CIL-EC-5380 for dl-PCBs, and CIL-EC-5385 for ndl-PCBs according to EPA Method 1613B^33^ and DIN EN 1948-4.^35^ For quality assurance, blanks, certified milk powder containing PCDD/Fs (BCR-607) and PCBs (BCR-450) and daily calibration checks were performed. Recoveries of the isotope-labeled congeners were within the limits set by EPA Method 1613B^33^ for the PCDD/Fs and EPA Method 1668C^34^ for the PCBs. The congener-specific limit of quantification (LOQ, Table S3) was calculated based on a signal-to-noise ratio of 3:1.^36^ In sample analysis, all values below the LOQ were reported as half of the respective LOQ value.

#### Estimation of Transfer Rates (TRs)

The TRs describe the percentual excretion via milk compared to the daily intake of a substance. In this study, the intake of contaminants occurred mainly through a daily bolus. Therefore, the congener-specific TRs can be calculated as
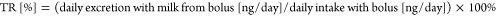
1To estimate the amount excreted with milk that came from bolus, a background correction was conducted for PCDD/Fs and PCB content in milk fat of the experimental group. Therefore, the average congener-specific content in milk fat of the control cows during NEB (1–56 DIM) and PEB (179–234 DIM), was subtracted from the concentration analyzed in the experimental group during the respective phases. Excretion of congeners from the first dosing during the second dosing was found to be negligible since the milk fat prior to the second dosing only had very low concentrations.

Before the steady state is reached, the congener concentrations in milk fat and thus the TRs keep increasing over weeks or even months. Therefore, TRs should be reported including the time frame from which they are derived, or alternatively they should be extrapolated to (near) steady-state conditions, often 90% of steady state. The TRs reported in this work are averaged over the last week of exposure including the first day of the depuration phase.

#### Evaluation of Cow Energy Balance

The chain length of fatty acids in milk fat indicates their origin, as the short- to medium-chain fatty acids C4:0–C14:0 stem from de novo synthesis in the mammary gland, while long-chain fatty acids (LCFA; C18:0–C24:0) are assimilated from feed or released during body fat mobilization.^37^ Only C16:0 may originate from de novo synthesis, mobilization of body fat, and/or from feed.^38,39^ Therefore, the percentage of LCFAs in milk fat increases when the cow mobilizes body fat at the beginning of the lactation to fulfill the high energy requirements, and decreases in the later NEB with increasing feed intake. During the PEB phase, the milk fatty acid composition is dominated by short- and medium-chain fatty acids from de novo synthesis with a nearly constant percentage of LCFAs. This biphasic LCFA trend in milk was evaluated using a broken line regression model^40^ to determine the end of NEB as the end of LCFA decrease in milk. The LCFAs included all saturated and unsaturated fatty acids with eighteen or more carbon atoms.

The fatty acid composition was measured in pooled milk samples with gas chromatography-flame ionization detection (GC-FID)^41^ for each cow once a week. For this, milk of four consecutive milkings was homogenized at 38 °C in a water bath and pooled according to the individual milk yield of the sample date. From these pooled samples, fat was extracted according to the Röse-Gottlieb method.^42^ Separation and quantification of fatty acids were conducted using a 7890A GC (Agilent Technologies, Santa Clara, CA) with a split injection port (ratio 1:100) and a 100 m CP-Sil 88 column (Agilent Technologies) after transesterification of triglycerides into fatty acid methyl esters (FAME).^41^ A constant flow of 1.3 mL/min hydrogen was used as the carrier gas. For analysis, the initial oven temperature was held at 60 °C for 1 min, increased by 2 °C/min to 105 °C (held for 1 min), increased by 35 °C/min to 172 °C, followed by an increase of 1 °C/min to 190 °C and a further increase of 5 °C/min to 225 °C (held for 10 min), and concluded by a final increase of 3 °C/min to 237 °C. Evaluation of chromatograms was performed by EZChrom Elite 3.3.2 (Agilent Technologies). The system was calibrated for fatty acids C4:0 to C24:0 using the reference milk fat CRM 164 (IRMM, Geel, Belgium). Fatty acid contents were calculated as weight percentage (g FA/100 g FA).

#### Statistics

Statistical analysis of the data was carried out using the statistical analysis package SAS (version 9.4, SAS Institute, Cary, NC). The considered analytes in milk were processed using the PROC MIXED procedure containing energy balance phases as fixed factor (negative (NEB)/positive-(PEB)). Because of repeated measurements during the study, individual animal effects were considered by using REPEATED and RANDOM statement in the procedure, fitting for a correlation structure within the animals that decreases with increasing lag between measurements, resulting in the calculated least squares means (LSmeans) with standard error of the means (±SEM). A tendency or low significance was defined for *p* < 0.1, whereas a significance was considered for *p* < 0.05 and a high significance for *p* < 0.01.

The Wilcoxon signed-rank test, as a nonparametric statistical test, was used in the context of the study, to test the calculated TRs of the congeners against literature data, with the null hypothesis being a specific value (LSmeans of the congeners). The test involved ranking the absolute differences between the observations and calculating the test statistic based on the sum of the ranks of positive and negative differences.

## Results and Discussion

### Performance Data and Energy Balance

Individual average animal performance data and body weights during the respective last week of the two dosing periods were recorded, and the LSmeans for the experimental and control groups were calculated (Table 3). In both groups, the body weight was significantly higher (*p* < 0.01) during the PEB phase, while milk and milk fat yields were significantly higher (*p* < 0.01) during the NEB phase, which corresponds with the expected course of milk yield (Figures S2–S10) and body weight (Figures S11–S19) in high-yielding dairy cows.^43,44^

**Table 3:** Key and Performance Data of Cows during the Last Week of Dosing in Each of the Two Phases^a^,^b^

		average values during phase 1: 22–29 days p.p. negative energy balance (NEB)				average values during phase 2: around 200–207 days p.p. positive energy balance (PEB)				
group	cow ID	body weight [kg]	milk [kg/day]	milk fat [kg/day]	milk fat [%]	body weight [kg]	milk [kg/day]	milk fat [kg/day]	milk fat [%]	transition week between NEB and PEB [WOL]
experimental	3426	654 ± 11 (8)	42.8 ± 0.7 (8)	2.1 ± 0.1 (5)	4.9 ± 0.2 (5)	691 ± 8 (5)	41.5 ± 1.5 (8)	1.7 ± 0.1 (6)	4.1 ± 0.2 (6)	12
	3438	674 ± 5 (7)	47.9 ± 2.2 (8)	2.0 ± 0.1 (5)	4.1 ± 0.4 (5)	702 ± 4 (6)	39.0 ± 0.9 (8)	1.6 ± 0.1 (5)	4.2 ± 0.3 (5)	8
	3448	672 ± 3 (5)	42.2 ± 1.5 (8)	1.9 ± 0.2 (5)	4.5 ± 0.3 (5)	717 ± 4 (2)	31.1 ± 2.0 (8)	1.4 ± 0.1 (6)	4.8 ± 0.1 (6)	22
	LSmeans	667 ± 6^d^	44.3 ± 1.8^d^	2.0 ± 0.1^d^	4.5 ± 0.2	702 ± 6^d^	37.2 ± 1.9^d^	1.6 ± 0.1^d^	4.4 ± 0.2	
	3425^e^	644 ± 9 (3)	45.6 ± 1.4 (8)	2.0 ± 0.2 (6)	4.4 ± 0.3 (6)	635 ± 5 (3)	38.4 ± 1.3 (8)	1.7 ± 0.1 (5)	4.5 ± 0.2 (5)	15
	3441^e^	642 ± 8 (6)	44.6 ± 8.2 (8)	1.6 ± 0.1 (5)	4.0 ± 0.9 (5)	died				18
control	3419	655 ± 8 (7)	47.3 ± 2.2 (8)	1.9 ± 0.1 (3)	4.1 ± 0.3 (3)	678 ± 11 (5)	44.7 ± 1.2 (8)	1.4 ± 0.2 (2)	3.3 ± 0.6 (2)	13
	3420^c^	598 ± 9 (6)	46.6 ± 1.9 (8)	no data available		641 ± 2 (4)	31.9 ± 1.1 (8)	1.3 ± 0.1 (4)	4.2 ± 0.1 (4)	11
	3432	658 ± 8 (8)	44.2 ± 1.8 (8)	1.7 ± 0.1 (2)	4.0 ± 0.3 (2)	719 ± 9 (8)	34.3 ± 0.9 (8)	1.4 ± 0.1 (3)	4.3 ± 0.1 (3)	28
	LSmeans	636 ± 19.8^d^	46.0 ± 1.5^d^	1.8 ± 0.1^d^	4.0 ± 0.2	682 ± 20^d^	37.0 ± 1.6^d^	1.4 ± 0.1^d^	4.0 ± 0.2	
	3434^e^	639 ± 12 (4)	46.3 ± 3.7 (8)	2.1 ± 0.1 (4)	4.3 ± 0.2 (4)	died				9

a Values in parentheses depict the number of observations.

b Individual cow data is represented as the arithmetic mean with standard deviation and group data as LSmeans with SEM.

c Replacement for cow 3434.

d Marks LSmeans with statistically significant differences (*p* < 0.01) between both phases within the experimental and control group.

e Cows excluded from statistical analyses; the exclusion criteria are explained under the section Clinical Findings in Cows 3434 and 3441, and the section PCDD/Fs and PCBs in Feces and Exclusion of Cow 3425.

The transition point from NEB to PEB was estimated for all nine cows based on the analysis of fatty acids in milk fat and ranged from week of lactation (WOL) 9 to 28 in the control group and WOL 8 to 22 in the experimental group (Table 3 and Figures S20–S28). Therefore, all experimental cows were in a PEB when the second dosing period was started around WOL 26.

### Clinical Findings in Cows 3434 and 3441

During the trial, control cow 3434 and experimental cow 3441 died in the course of a chronic metabolic disease. Both cows had a high initial milk production, with an average of, respectively, 46.8 kg/day (3434) and 44.9 kg/day (3441) within the second week of lactation, and showed increased blood β-hydroxybutyrate values indicative of ketosis. They proceeded to develop a chronic, therapy-resistant diarrhea and died 22–28 weeks postpartum. The control cow 3434 was replaced by another control cow in mid lactation (cow 3420).

The animals were dissected and examined pathologically by a specialized veterinarian. Both cows were diagnosed with hepatic lipidosis, which is one of the main health disorders in early lactating dairy cows.^45^ According to the literature, such high-yielding dairy cows are especially prone to develop this disease, as their feed intake can hardly cover their high energy requirements for milk production at the beginning of lactation.^46^ As part of the resulting lipid mobilization, triglycerides are deposited in liver cells, leading to the typical increased liver fat content.^47,48^ Furthermore, both cows were diagnosed with inflammation of the gastrointestinal tract, explaining their chronic, therapy-resistant diarrhea. The gastrointestinal inflammation was possibly caused by a subacute ruminal acidosis (SARA) which is a very common disease in high-yielding dairy cows leading to digestive disorders.^49^ However, based on the results of the dissection, the etiology could not be clarified conclusively. Although the mean retention time of feed through the gastrointestinal tract of a cow is around 2 days,^50^ diarrhea can lead to shorter digesta retention times and thus likely reduce PCDD/F and PCB absorption. Due to the clinical diagnosis and death before the PEB dosing regimen, data from cow 3441 and cow 3434 were not included in the following statistical assessments comparing NEB and PEB.

### PCDD/Fs and PCBs in Feed

Like most persistent organic pollutants, PCDD/Fs and especially PCBs are ubiquitous in background amounts in the environment. Therefore, the PCDD/F and PCB content in feed and milk of the control cows was analyzed on a regular basis. Only 4 of the 17 PCDD/Fs were detected in most feed samples (1,2,3,4,6,7,8-HpCDD, OCDD, 1,2,3,4,6,7,8-HpCDF, and OCDF). Other PCDD/Fs were detected only sporadically. Most ndl- and dl-PCBs were detectable in all feed materials, with the highest amounts in the mixed grass and maize silage. The exceptions were PCB-123, which was not detected at all, and PCB-114, which was not found in the samples of concentrate feed. Overall, the mean total TEQ content was 0.11 ng WHO_2005_-PCDD/F-PCB-TEQ/kg (88% dry matter, DM) in the grass and maize silage mixture, and 0.03 ng WHO_2005_-PCDD/F-PCB-TEQ/kg (88% DM) in the concentrates. Mean ndl-PCB (sum of the 6 indicator PCBs) content was 1.17 μg/kg (88% DM) in the grass and maize silage mixture and 0.52 μg/kg (88% DM) in the concentrate feed. These TEQ and ndl-PCB values are below EU maximum level (ML)^6^ and within expected ranges for animal feed in Germany.^51^

### PCDD/Fs and PCBs in Feces and Exclusion of Cow 3425

During both dosing periods, the mean WHO_2005_-PCDD/F-PCB-TEQ concentration in feces was calculated for each experimental cow based on the individual fecal samples from day 7 of the dosing regimen until 1 day after the end of the dosing (day 29).

**Figure 1:**
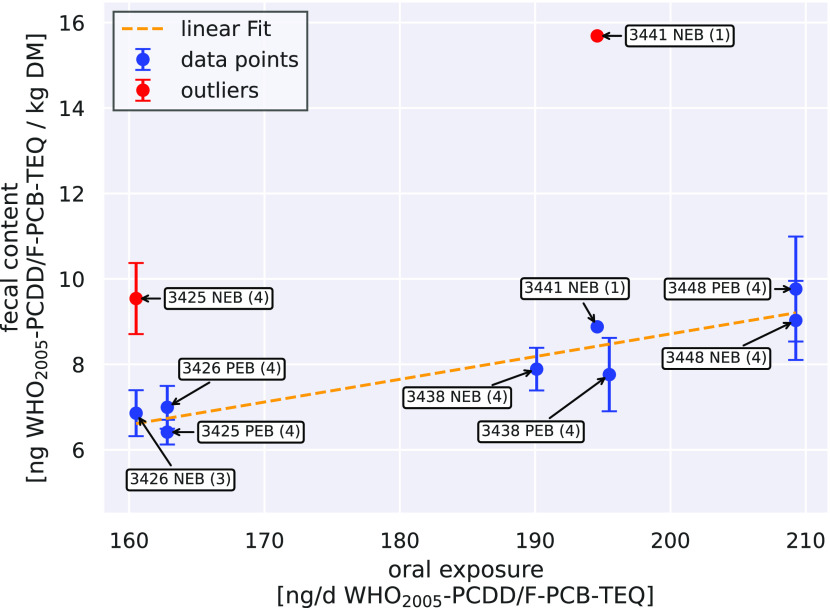
Mean WHO_2005_-PCDD/F-PCB-TEQ content in DM feces in relation to the daily dosage during the second to fourth week of the first dosing regimen. Numbers in parentheses indicate the amount of observations. During the NEB phase, WHO_2005_-PCDD/F-PCB-TEQ content in fecal DM of cow 3425 (red circle filled) (over 4 weeks) and cow 3441 (blue circle filled) (1 day) were elevated compared to the other cows.

As Figure 1 illustrates, the WHO_2005_-PCDD/F-PCB-TEQ concentration in feces based on DM was overall proportional to the daily bolus dosage (linear regression: *R*^2^ = 0.86; *R* = 0.93). As the daily amount of feces was not determined in this study, no statements can be made about the overall daily excretion of PCDD/Fs via feces. Nevertheless, the proportionality of feces concentration to the daily dosage is fitting with the observations of Richter and McLachlan, who described a correlation between daily uptake and fecal excretion of PCDD/Fs.^26^

However, during the NEB phase, cows 3425 and 3441 deviated from this correlation (Figure 1) with elevated fecal WHO_2005_-PCDD/F-PCB-TEQ content compared to the other experimental cows on multiple occasions. WHO_2005_-PCDD/F-PCB-TEQ content was elevated in all four fecal samples of cow 3425 during the NEB dosing phase, which correlated with an elevated fecal crude fat content (4–6% in DM, Table S4 and Figure S29). These elevated fecal crude fat contents were determined as outliers by Dixon-, Grubbs- and Mandel’s h-tests (95% significance level) in comparison to the other cows, which showed on average 1–3% in DM during the NEB phase. Elevated WHO_2005_-PCDD/F-PCB-TEQ and fecal crude fat content (6% in DM) for cow 3441 were only observed on day 29 postpartum.

Based on the experimental data, the etiology of the increased fecal crude fat concentrations in these two cows could not be answered definitely. We hypothesize a malabsorption of dietary lipids which could have been caused by different grades of SARA.

During the PEB dosing regimen, the fecal crude fat content of cow 3425 decreased (average 3% in DM) and WHO_2005_-PCDD/F-PCB-TEQ content in feces no longer deviated from the linear regression (Figure 1). These observations indicate a positive correlation of the fecal crude fat and the WHO_2005_-PCDD/F-PCB-TEQ content in fecal DM. To date, there are no data available on this correlation in ruminants, but there is experimental evidence that unabsorbable fat supplements in diets increase fecal excretion of lipophilic contaminants such as 2,3,7,8-TCDD in humans^52,53^ or 2,2′,4,4′-tetrabromodiphenyl ether in male Wistar rats.^54^

Fecal WHO_2005_-PCDD/F-PCB-TEQ content was on average 0.07 ng/kg DM for the control cows during the same time frame in the NEB and PEB phases, respectively. Fecal crude fat content was on average 1–3% in DM.

### PCDD/Fs and PCBs in Milk

In the milk of the control cows (3419, 3420, and 3432), ndl-PCBs (mean ∑-ndl-PCBs 2.8 ± 1.9 ng/g milk fat) and most dl-PCBs (mean 0.25 ± 0.06 pg WHO_2005_-PCB-TEQ/g milk fat) were found in all samples during the lactation cycle. The high variation of the ndl-PCBs was mostly due to the lower-chlorinated PCB-28, -52, and -101. The WHO_2005_-PCB-TEQ content in milk fat constantly dropped during the lactation cycle from 0.29 ± 0.03 pg WHO_2005_-PCB-TEQ/g milk fat (DIM 1–56) to 0.20 ± 0.02 pg WHO_2005_-PCB-TEQ/g milk fat (DIM 176–233). It is possible that PCBs accumulated during the dry phase and were mobilized with body fat after calving. However, this would follow a biphasic depuration with an initial drop due to fast elimination followed by slower elimination^55−60^ that was not observed here. Differences in background levels were nevertheless taken into account by using separate background corrections for the NEB and PEB phases to derive TRs. Most PCDD/Fs were rarely detected in controls (mean 0.05 ± 0.03 pg WHO_2005_-PCDD/F-TEQ/g milk fat). These background levels are well below current EU regulation limits^5,7^ and are typical for dairy products on the German market.^61,62^

**Figure 2:**
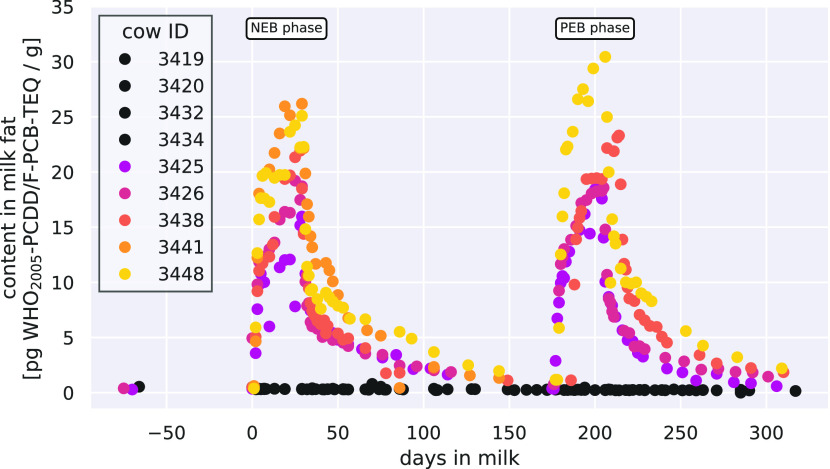
Content in milk fat as the WHO_2005_-PCDD/F-PCB-TEQ sum, trend for the experimental and control cows during the NEB and PEB phases of lactation. Black dots are control cows, while colored dots are experimental cows.

In milk fat samples of the experimental group, most congeners showed a distinctive increase during the dosing phases and a decrease during the depuration phases (Figure 2). This was less pronounced for OCDD and OCDF and was not observable for the low-chlorinated indicator ndl-PCBs (PCB-28, -52, and -101) due to highly fluctuating background values. Therefore, these three ndl-PCBs were excluded from further evaluations of congener-specific TRs.

As expected, the highest levels of congeners in milk fat were found for cow 3448, which received the highest daily dosages (Table 2). In general, the congener levels in milk fat increased rapidly with the beginning of the dosage regimen. During the end of the dosing phase, most congener-specific levels in milk fat appeared to still be increasing (should the dosing be extended). Based on these observations, it is unlikely that all congeners reached a steady state at the end of the two dosing phases. Only three congeners with fast transfer kinetics, namely, 2,3,7,8-TCDF, 1,2,3,7,8-PeCDF, and PCB-77, might have reached a steady state based on the observed levels in milk fat. Furthermore, at the end of the exposure phase congener profiles in milk were different compared to the daily dosage, whereas the profiles of the daily dosage and feces were similar (Figures S30–S32 for cow 3426, 3438, and 3448).

During the initial depuration phases, the milk fat content decreased rapidly in the first week, and slower thereafter. Shortly before the second dosing phase WHO_2005_-PCDD/F-PCB-TEQ content in milk fat nearly reached levels observed in the control cows.

### Transfer Rates (TRs)

**Figure 3:**
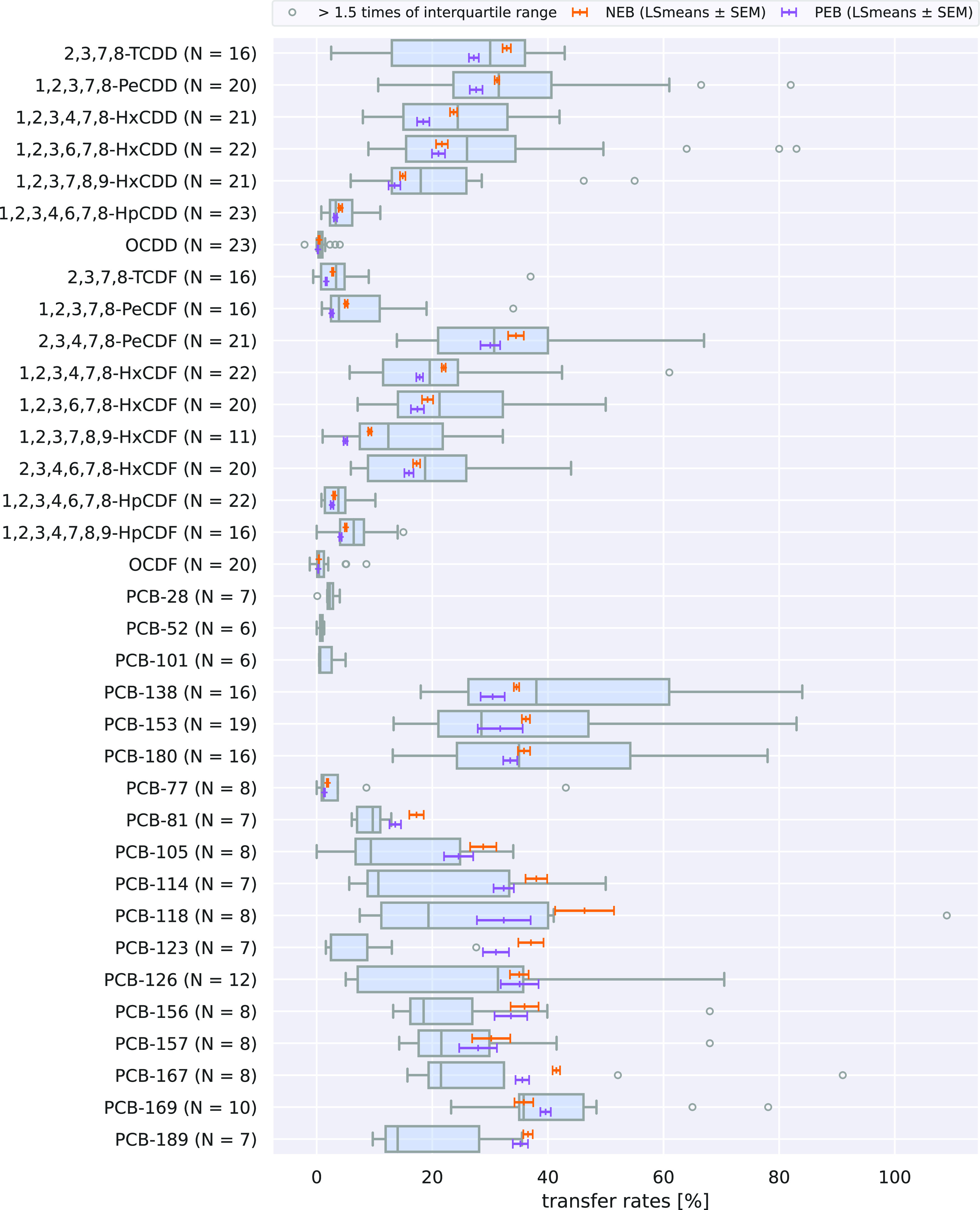
Transfer rates (TRs) from this study (horizontal colored lines) compared to literature data (box plots and circles). The lines show LSmeans ± SEM from this study in the negative energy balance (NEB, orange) and positive energy balance (PEB, purple) based on cows 3426, 3441, and 3448 (Tables 4 and 5). Box plots and circles show literature data,^9^ where the boxes (*N* > 5) are defined as the interquartile range (IQR) between 25th (Q1)–75th (Q3) percentiles of the data, and the gray line in the box represents the median; Whiskers include data within 1.5 times of IQR below Q1 and above Q3. Plot generated with Python 3.10 using the Seaborn, Matplotlib, Numpy, and Pandas libraries.

Congener-specific TRs are presented in Figure 3 and for each experimental cow in Tables S5 and S6. As hypothesized, most cow individual congener-specific TRs tend to be higher during the first dosing (NEB) compared to the second dosing (PEB). However, there were some features of the data that need to be discussed. One exception were the TRs of cow 3425, where 20 of the 32 analyzed congeners were lower during the NEB phase compared to the PEB phase (Tables S9 and S10).

Furthermore, 15 of these NEB phase TRs of cow 3425 were the lowest congener-specific TRs observed for all experimental cows during both phases. This observation for cow 3425 correlates with its aforementioned high fecal crude fat content during the NEB phase as well as its elevated WHO_2005_-PCDD/F-PCB-TEQ content in feces (Figure 1) and lower WHO_2005_-PCDD/F-PCB-TEQ content in milk fat (Figure 2) compared to cow 3426 which received the same daily dosage (Table 2). This strengthens the hypothesis that an elevated undigested fat content in feces reduces intestinal absorption and therefore lowers the amount of contaminants transferred into milk. TRs derived for cow 3441 were not noticeably affected by diarrhea on day 29 of the first dosing phase. However, due to the chronic health problems of cow 3441, subclinical influences on the transfer cannot be ruled out. These observations confirm the decision to exclude experimental cows 3425 and 3441 from further statistical assessments comparing metabolic phases.

### Statistical Evaluation of the Transfer Rates

Based on the aforementioned criteria to compare TRs derived from the same healthy cows during NEB and PEB, the statistical evaluation was carried out with cows 3426, 3438, and 3448. The LSmeans were calculated and statistically evaluated for these animals using a mixed model.

Table 4 shows the results of the statistical evaluation for the PCDD/F TRs. In addition, total TRs over both phases (NEB and PEB) were calculated. Comparing both dosing periods, overall fourteen of the seventeen tested congeners (82%) showed statistically higher TRs during the NEB phase (53% *p* < 0.01, 18% *p* < 0.05, 12% *p* < 0.1). However, the TRs of three congeners (1,2,3,6,7,8-HxCDD, 1,2,3,7,8,9-HxCDD, and 1,2,3,4,6,7,8-HpCDF) did not differ significantly between NEB and PEB.

**Table 4:** Statistical Evaluation of PCDD/F Transfer Rates (TRs) from Cow 3426, 3438, and 3448 Using a Mixed Model

	NEB TRs		PEB TRs		difference of PEB-NEB		total TRs (NEB and PEB)	
	LSmeans	SEM	LSmeans	SEM	δ	*p* value	LSmeans	SEM
2,3,7,8-TCDD^b^	32.87	0.71	27.19	0.85	5.68	<0.001	29.85	0.82
1,2,3,7,8-PeCDD^c^	31.18	0.40	27.58	1.10	3.60	0.040	29.12	0.81
1,2,3,4,7,8-HxCDD^c^	23.68	0.61	18.43	1.07	5.25	0.012	20.83	0.88
1,2,3,6,7,8-HxCDD	21.67	1.02	21.07	1.13	0.60	0.491	21.28	0.91
1,2,3,7,8,9-HxCDD	14.88	0.47	13.46	1.02	1.42	0.310	13.68	0.65
1,2,3,4,6,7,8-HpCDD^b^	4.11	0.30	3.24	0.27	0.87	0.004	3.78	0.30
OCDD^a^,^d^	0.41	0.11	0.20	0.05	0.21	0.072	0.29	0.08
2,3,7,8-TCDF^b^	2.77	0.13	1.65	0.12	1.11	<0.001	2.21	0.29
1,2,3,7,8-PeCDF^b^	5.11	0.25	2.59	0.24	2.52	<0.001	3.95	0.49
2,3,4,7,8-PeCDF^b^	34.47	1.36	30.03	1.70	4.44	0.005	32.35	1.42
1,2,3,4,7,8-HxCDF^b^	21.97	0.34	17.81	0.58	4.16	0.001	19.74	0.83
1,2,3,6,7,8-HxCDF^c^	19.18	0.96	17.42	1.12	1.75	0.027	18.30	1.13
1,2,3,7,8,9-HxCDF^b^	9.19	0.28	4.97	0.31	4.22	<0.001	7.17	0.76
2,3,4,6,7,8-HxCDF^d^	17.28	0.61	15.96	0.79	1.32	0.095	16.41	0.58
1,2,3,4,6,7,8-HpCDF	3.02	0.17	2.66	0.24	0.37	0.155	2.83	0.24
1,2,3,4,7,8,9-HpCDF^b^	5.03	0.16	4.13	0.21	0.90	0.004	4.57	0.18
OCDF^b^	0.37	0.02	0.27	0.02	0.10	0.003	0.32	0.02

a Marks congeners that were also present in method blanks.

b Statistical evidence is strong (*p* < 0.01) for NEB TR > PEB TR.

c Statistical evidence is medium (*p* < 0.05) for NEB TR > PEB TR.

d Statistical evidence is weak (*p* < 0.1) for NEB TR > PEB TR.

Regarding the twelve tested dl-PCBs (Table 5), the TRs of seven congeners (58%) were significantly higher during the NEB phase (33% *p* < 0.01, 17% *p* < 0.05, 8% *p* < 0.1). TRs for PCB-169 were statistically higher during the PEB phase (*p* < 0.1). The TR of the other four dl-PCB congeners (126, 156, 157, and 189) as well as the three ndl-PCBs showed no significant differences between the dosing periods. The statistical difference between NEB and PEB was less pronounced for the PCBs compared to the PCDD/Fs, likely due to fluctuations of the aforementioned PCB background contamination observed in milk fat of the control group.

**Table 5:** Statistical Evaluation of PCB Transfer Rates (TRs) from Cow 3426, 3438, and 3448 Using a Mixed Model

	NEB TRs		PEB TRs		difference of PEB-NEB		total TRs (NEB and PEB)	
	LSmeans	SEM	LSmeans	SEM	δ	p value	LSmeans	SEM
PCB-138^a^	34.57	0.46	30.44	2.08	4.13	0.184	31.58	1.28
PCB-153^a^	36.19	0.70	31.75	3.89	4.44	0.378	32.93	1.74
PCB-180^a^	35.86	1.05	33.48	1.21	2.38	0.217	34.25	0.83
PCB-77^a^,^c^	1.88	0.13	1.33	0.10	0.55	0.039	1.61	0.18
PCB-81^d^	17.27	1.25	13.59	0.99	3.68	0.075	15.22	1.23
PCB-105^a^,^c^	28.81	2.27	24.55	2.52	4.26	0.028	26.75	2.61
PCB-114^b^	38.00	1.87	32.35	1.76	5.65	0.001	35.20	1.52
PCB-118^a^,^b^	46.34	5.09	32.36	4.65	13.98	0.002	38.52	5.09
PCB-123^b^	37.06	2.18	31.01	2.25	6.05	<0.001	34.10	2.02
PCB-126	35.04	1.61	35.11	3.27	–0.07	0.987	34.12	1.30
PCB-156^a^	35.96	2.42	33.59	2.83	2.37	0.186	34.96	2.87
PCB-157^a^	30.19	3.30	27.92	3.26	2.28	0.161	28.97	3.42
PCB-167^a^,^b^	41.45	0.64	35.57	1.18	5.88	0.008	38.02	1.15
PCB-169^e^	35.84	1.62	39.60	0.90	–3.76	0.094	37.33	1.55
PCB-189^a^	36.55	0.81	35.24	1.31	1.32	0.395	35.58	0.76

a Marks congeners that were also present in method blanks.

b Statistical evidence is strong (*p* < 0.01) for NEB TR > PEB TR.

c Statistical evidence is medium (*p* < 0.05) for NEB TR > PEB TR.

d Statistical evidence is weak (*p* < 0.1) for NEB TR > PEB TR.

e Statistical evidence is weak (*p* < 0.1) for PEB TR > NEB TR.

In summary, the TRs of 21 from the 32 analyzed PCDD/F and PCB congeners were statistically higher during the NEB phase indicating a distinct influence of the cows’ metabolic situation on the transfer of contaminants into milk. The higher TRs during the NEB phase correlate with the significantly higher daily milk fat production during the NEB phase (Table 3). Presumably, this favors the excretion of lipophilic substances with milk as has already been suggested by others.^13,63^ Furthermore, a possible effect of energy balance on the transfer of lipophilic contaminants due to changes of the body fat reservoirs was discussed before.^18,64−66^ This effect was suspected from observation^19,21,67^ and modeled in the literature,^68^ but has not been statistically validated yet. The present study shows that there is indeed a significant association between energy balance and transfer kinetics.

### Comparison of the Transfer Rates with Literature Data

As shown in Figure 3, most TRs calculated for NEB and PEB in the last week of a 4-week exposure phase are in the range of previously reported TRs derived from similar studies. This literature data set consists of congener-specific TRs from studies with dairy or dual-purpose cows, an oral exposure time of at least 28 days, and known congener inputs.^9^ Recently published transfer rates for some PCDD/Fs and PCBs^12,69^ are not included in this data set.

In detail, congener-specific TRs for the PCDD/Fs for NEB and PEB phase are below the 75th percentile of the literature data and, with the exception of PEB TRs for OCDD and 1,2,3,7,8,9-HxCDF, above the 25th percentile. Only five NEB TRs (2,3,7,8-TCDD, 1,2,3,4,6,7,8-HpCDD, 1,2,3,7,8-PeCDF, 2,3,4,7,8-PeCDF, and 1,2,3,4,7,8-HxCDF) are above median derived from literature data. Based on the Wilcoxon test, lower total TRs (NEB and PEB) of OCDD and 1,2,3,7,8,9-HxCDF are significant (*p* < 0.05) compared to the literature data.

TRs for the three ndl-PCBs are also within the 25th and 75th percentile of the literature data, with both TRs for PCB-138 below the median and for PCB-153 above the median. For PCB-180, the NEB TR is above the median and the PEB TR below the median. Again, no statistical differences for these congeners are evident between total TRs (NEB and PEB) and literature data.

The major differences to literature data were found for the dl-PCB TRs. TRs of only three congeners (PCB-77 PCB-126 and PCB-169) are between the 25th and 75th percentile of the literature data during the NEB and PEB phase, respectively. Of the twelve dl-PCBs, nine NEB TRs and five PEB TRs are above the 75th percentile. The difference of the elevated total TRs (NEB and PEB) for PCB-81, PCB-123, and PCB-189 is significant (*p* < 0.05) compared to the literature data. TRs of these three congeners are among the highest reported congener-specific TRs, with the total TRs (NEB and PEB) for PCB-123 (34.1%) being the highest reported yet. Nevertheless, the TR for PCB-123 is similar to the TR for dairy cows (27.6%) reported by Lorenzi et al.^15^ and a TR reported for dairy buffaloes (27.1%).^70^ The latter was not included in the data set by Krause et al. and therefore is also not included in Figure 3.^9^

All things considered, most congener-specific PEB TRs of this study are distributed between the 25th and 75th percentile of the literature data (Figure 3). NEB phase TRs tend to be higher and especially for most dl-PCBs these are above the 75th percentile. However, TRs from these compared studies also exhibited high variances. This mirrors the complexity of the transfer from oral exposure to milk; differences between studies seem to have more reasons than just the milk performance of the cows. Hence, factors like exposure time,^71^ bioavailability^72^ as well as the health^73^ and metabolic state^19,64^ need to be considered besides daily milk and milk fat production.

The data from this study from the selected three experimental and three control cows is the subject of a related publication,^74^ where TRs are also derived. Those TRs (Figure S1 and Table S3 of ref (74)) are obtained from toxicokinetic modeling and are extrapolated to the steady state, whereas those presented here are calculated directly from the data using eq 1 and averaged over the last week of exposure including the first day of the depuration phase. As steady-state condition was not reached within the 28-day dosing regimen, TRs derived from toxicokinetic modeling under steady-state conditions are higher than those presented here.

In conclusion, the TRs of seventeen PCDD/F, twelve dl-PCB, and three ndl-PCB into the milk of high-yielding dairy cows were statistically analyzed under two different metabolic conditions (NEB and PEB). For fourteen PCDD/Fs (82%), seven dl-PCBs (58%) but none of the ndl-PCB congeners, the TRs were significantly higher during the NEB compared to the PEB phase. This statistically significant association between metabolic state and transfer kinetics is shown here for the first time. Further research is needed to prove its causality and to understand the underlying mechanisms.

In comparison to the literature data, a tendency for elevated TRs was observed for dl-PCBs, especially during the NEB phase. However, only three congener-specific TRs were significantly higher. The congener-specific total TRs (NEB and PEB) derived for fifteen of the seventeen PCDD/Fs and the three ndl-PCBs were within the range of former studies with mainly low-yielding dairy cows. However, these transfer studies were conducted with a variety of different designs and revealed a wide range of congener-specific TRs, impeding the comparability of data and their use for mathematical modeling. In the present study, the transfer of all relevant PCDD/F and dl-PCB congeners was analyzed at once for the first time in high-yielding dairy cows, ensuring ideal comparability among congener-specific TRs. The data from this transfer study can be used to develop and refine toxicokinetic models for risk assessment of high-yielding dairy cows.

## Supporting Information Available

Experimental details, dosing regimen, sampling scheme, limit of quantification, performance parameters of the cows, body weight, distribution of long-chain fatty acids in milk, fecal crude fat, PCDD/F and PCB content in milk fat, transfer rates, and congener profiles (PDF)

## Abbreviations

BW: body weight
DIM: days in milk
DM: dry mass
GC: gas chromatograph
HRMS: high-resolution mass spectrometer
HpCDD: heptachlorodibenzo-*p*-dioxin
HpCDF: heptachlorodibenzofuran
HxCDD: hexachlorodibenzo-*p*-dioxin
HxCDF: hexachlorodibenzofuran
IQR: interquartile range
LCFA: long-chain fatty acids
LOQ: limit of quantification
LSmeans ± SEM: least squares means with standard error of the means
ML: maximum level
NEB: negative energy balance
OCDD: octachlorodibenzo-*p*-dioxin
OCDF: octachlorodibenzofuran
PCBs: polychlorinated biphenyls
PCDD/Fs: polychlorinated dibenzo-*p*-dioxins and dibenzofurans
PCDDs: polychlorinated dibenzo-*p*-dioxins
PCDFs: polychlorinated dibenzofurans
PEB: positive energy balance
POPs: persistent organic pollutants
PeCDD: pentachlorodibenzo-*p*-dioxin
PeCDF: pentachlorodibenzofuran
SARA: subacute ruminal acidosis
TCDD: tetrachlorodibenzo-*p*-dioxin
TCDF: tetrachlorodibenzofuran
TEF: toxic equivalency factor
TEQ: toxic equivalency
TR: transfer rate
WHO: World Health Organization
WOL: week of lactation
dl: dioxin-like
ndl: non-dioxin-like
