# Supplementary material for: Experimental Study on the Transfer of Polychlorinated Biphenyls (PCBs) and Polychlorinated Dibenzo-p-dioxins and Dibenzofurans (PCDD/Fs) into Milk of High-Yielding Cows during Negative and Positive Energy Balance
Source: J Agric Food Chem. 2023 Aug 31;71(36):13495–507. doi: 10.1021/acs.jafc.3c02776 (PMC10510706; doi:10.1021/acs.jafc.3c02776)
Supplement: Supplementary file 1 — jf3c02776_si_001.pdf [file jf3c02776_si_001.pdf]

Supporting Information

Experimental Study on the Transfer of Polychlorinated Biphenyls (PCBs) and Polychlorinated Dibenzo-*p*-dioxins and Dibenzofurans (PCDD/Fs) into Milk of High-Yielding Cows during Negative and Positive Energy Balance

Torsten Krause[1], Julika Lamp[1], Karin Knappstein[1], Hans-Georg Walte[1], Jan-Louis Moenning[2], Joachim Molkentin[1], Florian Ober[1], Andreas Susenbeth[3], Edwin Westreicher-Kristen[3], Karl-Heinz Schwind[4], Sven Dänicke[5], Peter Fürst[6], Hans Schenkel[7], Robert Pieper[2], Jorge Numata[2]

[1]Department of Safety and Quality of Milk and Fish Products, Max Rubner-Institut (MRI), Hermann-Weigmann-Str. 1, 24103 Kiel, Germany

[2]Department Safety in the Food Chain, German Federal Institute for Risk Assessment (BfR), Max-Dohrn-Straße 8-10, 10589 Berlin, Germany

[3]Institute of Animal Nutrition and Physiology, Kiel University (CAU), 24118 Kiel, Germany

[4]Department of Quality and Safety of Meat, Max Rubner-Institut (MRI), E.-C.-Baumann-Str. 20, 95326 Kulmbach, Germany

[5]Institute of Animal Nutrition, German Federal Research Institute for Animal Health, Friedrich-Loeffler-Institut (FLI), Bundesallee 37, 38116 Braunschweig, Germany

[6]Institute of Food Chemistry, University of Münster, Corrensstrasse 45, 48149 Münster, Germany

[7]Department of Animal Nutrition, University of Hohenheim, Emil-Wolff-Str. 10, 70599 Stuttgart, Germany

Corresponding author: jorge.numata@bfr.bund.de

Dosing regimen

Table S1. Individual dosage of PCDD/Fs and PCBs during the first experimental phase.

congener	product code	cow 3425 [ng/d]	cow 3426 [ng/d]	cow 3438 [ng/d]	cow 3441 [ng/d]	cow 3448 [ng/d]
2,3,7,8-TCDD	U-RPE-029S	17.00	17.00	20.00	20.00	20.88
1,2,3,7,8-PeCDD	CIL-ED-950	26.91	26.91	31.00	31.00	34.63
1,2,3,4,7,8-HxCDD	CIL-ED-961	40.50	40.50	47.67	47.67	52.13
1,2,3,6,7,8-HxCDD	CIL-ED-960	29.80	29.80	34.17	34.17	38.63
1,2,3,7,8,9-HxCDD	CIL-ED-969	44.25	44.25	52.83	52.83	56.79
1,2,3,4,6,7,8-HpCDD	CIL-ED-971	448.09	448.09	529.00	529.00	584.13
OCDD	CIL-ED-980-C	4418.00	4418.00	3378.00	3378.00	4529.57
2,3,7,8-TCDF	CIL-EF-903	326.82	326.82	398.67	398.67	441.13
1,2,3,7,8-PeCDF	CIL-EF-953	409.27	409.27	473.17	473.17	536.13
2,3,4,7,8-PeCDF	CIL-EF-956	23.00	23.00	23.80	23.80	28.00
1,2,3,4,7,8-HxCDF	CIL-EF-964	46.50	46.50	54.17	54.17	59.63
1,2,3,6,7,8-HxCDF	CIL-EF-962	31.10	31.10	34.50	34.50	38.75
1,2,3,7,8,9-HxCDF	CIL-EF-967	45.40	45.40	51.67	51.67	59.38
2,3,4,6,7,8-HxCDF	CIL-EF-968	48.90	48.90	55.00	55.00	62.13
1,2,3,4,6,7,8-HpCDF	CIL-EF-973	248.82	248.82	311.67	311.67	327.50
1,2,3,4,7,8,9-HpCDF	CIL-EF-975	111.27	111.27	135.50	135.50	149.13
OCDF	U-RPE-019S	1700.00	1700.00	1741.67	1741.67	1927.57
PCB-28	U-RPC-084S	3438.00	3438.00	4297.33	7040.67	7040.67
PCB-52	U-RPC-031S	5547.60	5547.60	7356.00	9761.33	9761.33
PCB-101	U-RPC-039S	10011.20	10011.20	13405.00	12628.67	12628.67
PCB-138	U-RPC-088S	10648.67	10648.67	13225.67	14797.00	14797.00
PCB-153	U-RPC-047S	8019.60	8019.60	9576.67	10377.00	10377.00
PCB-180	U-RPC-094S	2063.40	2063.40	2829.67	3114.00	3114.00
PCB-77	U-RPC-036S	5313.80	5313.80	6580.00	6684.67	6684.67
PCB-81	U-RPC-096S	1201.40	1201.40	1467.00	1354.00	1354.00
PCB-105	U-RPC-098S	762.60	762.60	928.67	1015.67	1015.67
PCB-114	U-RPC-108S	496.60	496.60	604.67	624.67	624.67
PCB-118	U-RPC-106S	1512.20	1512.20	1756.00	1600.67	1600.67
PCB-123	U-RPC-156S	2455.40	2455.40	2863.60	2843.00	2843.00
PCB-126	U-RPC-102S	323.20	323.20	396.67	438.33	438.33
PCB-156	U-RPC-055S	1130.00	1130.00	1372.00	1347.67	1347.67
PCB-157	U-RPC-164S	862.80	862.80	1074.67	1189.67	1189.67
PCB-167	U-RPC-100S	937.00	937.00	1288.00	1197.00	1197.00
PCB-169	U-RPC-090S	272.00	272.00	336.00	347.33	347.33
PCB-189	U-RPC-137S	1267.60	1267.60	1738.00	1569.00	1569.00

Table S2. Individual dosage of PCDD/Fs and PCBs during the second experimental phase.

congener	product code	cow 3425 [ng/d]	cow 3426 [ng/d]	cow 3438 [ng/d]	cow 3441 [ng/d]	cow 3448 [ng/d]
2,3,7,8-TCDD	U-RPE-029S	18.33	18.33	20.24	-	20.88
1,2,3,7,8-PeCDD	CIL-ED-950	25.50	25.50	29.65	-	34.63
1,2,3,4,7,8-HxCDD	CIL-ED-961	42.17	42.17	48.35	-	52.13
1,2,3,6,7,8-HxCDD	CIL-ED-960	28.67	28.67	37.20	-	38.63
1,2,3,7,8,9-HxCDD	CIL-ED-969	46.08	46.08	52.07	-	56.79
1,2,3,4,6,7,8-HpCDD	CIL-ED-971	463.83	463.83	584.40	-	584.13
OCDD	CIL-ED-980-C	5963.55	5963.55	6004.61	-	4529.57
2,3,7,8-TCDF	CIL-EF-903	344.17	344.17	419.67	-	441.13
1,2,3,7,8-PeCDF	CIL-EF-953	403.33	403.33	524.43	-	536.13
2,3,4,7,8-PeCDF	CIL-EF-956	20.83	20.83	24.11	-	28.00
1,2,3,4,7,8-HxCDF	CIL-EF-964	43.67	43.67	59.05	-	59.63
1,2,3,6,7,8-HxCDF	CIL-EF-962	31.83	31.83	38.00	-	38.75
1,2,3,7,8,9-HxCDF	CIL-EF-967	49.33	49.33	55.67	-	59.38
2,3,4,6,7,8-HxCDF	CIL-EF-968	48.83	48.83	58.95	-	62.13
1,2,3,4,6,7,8-HpCDF	CIL-EF-973	288.67	288.67	291.44	-	327.50
1,2,3,4,7,8,9-HpCDF	CIL-EF-975	101.00	101.00	134.00	-	149.13
OCDF	U-RPE-019S	1180.00	1180.00	1664.45	-	1927.57
PCB-28	U-RPC-084S	4954.33	4954.33	5017.00	-	7040.67
PCB-52	U-RPC-031S	6922.00	6922.00	8970.83	-	9761.33
PCB-101	U-RPC-039S	10841.33	10841.33	11404.00	-	12628.67
PCB-138	U-RPC-088S	11693.67	11693.67	12865.33	-	14797.00
PCB-153	U-RPC-047S	7818.67	7818.67	8674.67	-	10377.00
PCB-180	U-RPC-094S	2676.00	2676.00	2791.83	-	3114.00
PCB-77	U-RPC-036S	5174.00	5174.00	6960.17	-	6684.67
PCB-81	U-RPC-096S	1242.00	1242.00	1398.83	-	1354.00
PCB-105	U-RPC-098S	771.00	771.00	935.33	-	1015.67
PCB-114	U-RPC-108S	501.00	501.00	556.00	-	624.67
PCB-118	U-RPC-106S	1374.33	1374.33	1612.67	-	1600.67
PCB-123	U-RPC-156S	2210.00	2210.00	2800.50	-	2843.00
PCB-126	U-RPC-102S	331.33	331.33	413.00	-	438.33
PCB-156	U-RPC-055S	1070.00	1070.00	1290.17	-	1347.67
PCB-157	U-RPC-164S	877.33	877.33	1059.50	-	1189.67
PCB-167	U-RPC-100S	981.33	981.33	1180.00	-	1197.00
PCB-169	U-RPC-090S	260.67	260.67	321.33	-	347.33
PCB-189	U-RPC-137S	1253.67	1253.67	1577.33	-	1569.00

Sampling scheme

Day	-70	0	1	2	3	4	5	6	7	10	13	14	16	19	21	22	25	28	29	30	31	32	33	34
Week	-10		1							2			3			4			5					
			First dosing phase																First depuration phase					
Milk (residue analysis)	(x)	Calving	x	x	x	x	x	x	x	x	x		x	x		x	x	x	x	x	x	x	x	x
Milk (milk compound analysis)									x			x			x			x						
Milk (fatty acid analysis)									x			x			x			x						
Feed			x															x						
Feces			x						x		x			x					x					

Day	35	38	41	42	44	47	49	50	53	56	63	66	70	76	77	84	86	91	96	98	105	106	112	116	119	126	133	140	146	147	154	161	168	175	178
Week		6			7			8			9	10		11		12	13		14		15	16		17		18	19	20	21		22	23	24	25	26
Milk (residue analysis)	x	x	x		x	x		x	x	x		x		x			x		(x)			(x)		x		(x)			(x)						x
Milk (milk compound analysis)	x			x			x			x	x		x		x	x		x		x	x		x		x	x	x	x		x	x	x	x	x	
Milk (fatty acid analysis)	x			x			x			x	x		x		x	x		x		x	x		x		x	x	x	x		x	x	x	x	x	
Feed										x						x							x					x					x		
Feces																																			x

Day	179	180	181	182	183	184	185	188	189	191	194	196	197	200	203	206	207	208	209	210	211	212	213	216	217
Week	26				27					28			29			30	30				31				
	Second dosing phase																Second depuration phase								
Milk (residue analysis)	x	x	x	x	x	x	x	x		x	x			x	x	x	x	x	x	x	x	x	x	x	
Milk (milk compound analysis)				x					x			x			x					x					x
Milk (fatty acid analysis)				x					x			x			x					x					
Feed												x													
Feces							x			x			x				x								
Dissection and sampling																									

Day	219	222	224	225	228	231	234	238	244	245	252	254	259	264	266	273	274	280	284	287	294	301	304	305
Week	32			33			34		35		36	37		38		39	40		41		42	43	44	
Milk (residue analysis)	x	x		x	x	x	x		x			x		x			x		x		x		x	Euthanasia
Milk (milk compound analysis)			x			x		x		x	x		x		x	x		x		x	x	x		
Milk (fatty acid analysis)																								
Feed			x								x							x						
Feces																								
Dissection and sampling																								x

Figure S1. Scheme of dosing periods and sampling. Cells marked as (x) indicate samples that were not taken from all experimental cows. Milk for residue analysis were sampled less frequently in the control group.

Limit of quantification

Table S3. Limit of quantification (LOQ).

Matrix	instrumental LOQ on column (SN 3:1)	milk fat	feed (88% DM)	feces (DM)
Sample weight		5 g	10 g	10 g
Units	[fg]	[ng/kg]	[ng/kg]	[ng/kg]
2,3,7,8-TCDD	9	0.009	0.004	0.005
1,2,3,7,8-PeCDD	19	0.019	0.008	0.010
1,2,3,4,7,8-HxCDD	27	0.027	0.012	0.013
1,2,3,6,7,8-HxCDD	28	0.028	0.012	0.014
1,2,3,7,8,9-HxCDD	29	0.029	0.013	0.014
1,2,3,4,6,7,8-HpCDD	13	0.013	0.006	0.007
OCDD	20	0.020	0.009	0.010
2,3,7,8-TCDF	9	0.009	0.004	0.005
1,2,3,7,8-PeCDF	26	0.026	0.012	0.013
2,3,4,7,8-PeCDF	26	0.026	0.011	0.013
1,2,3,4,7,8-HxCDF	29	0.029	0.013	0.015
1,2,3,6,7,8-HxCDF	27	0.027	0.012	0.014
1,2,3,7,8,9-HxCDF	42	0.042	0.018	0.021
2,3,4,6,7,8-HxCDF	31	0.031	0.014	0.016
1,2,3,4,6,7,8-HpCDF	23	0.023	0.010	0.011
1,2,3,4,7,8,9-HpCDF	20	0.020	0.009	0.010
OCDF	20	0.020	0.009	0.010
PCB-28	12	0.048	0.021	0.024
PCB-52	4	0.016	0.007	0.008
PCB-101	21	0.084	0.037	0.042
PCB-138	7	0.026	0.012	0.013
PCB-153	7	0.030	0.013	0.015
PCB-180	5	0.019	0.008	0.009
PCB-77	24	0.095	0.042	0.048
PCB-81	21	0.083	0.037	0.042
PCB-105	33	0.131	0.058	0.065
PCB-114	31	0.123	0.054	0.062
PCB-118	28	0.111	0.049	0.056
PCB-123	31	0.124	0.055	0.062
PCB-126	46	0.184	0.081	0.092
PCB-156	12	0.048	0.021	0.024
PCB-157	13	0.050	0.022	0.025
PCB-167	16	0.066	0.029	0.033
PCB-169	15	0.059	0.026	0.030
PCB-189	7	0.030	0.013	0.015

Performance parameters of the cows

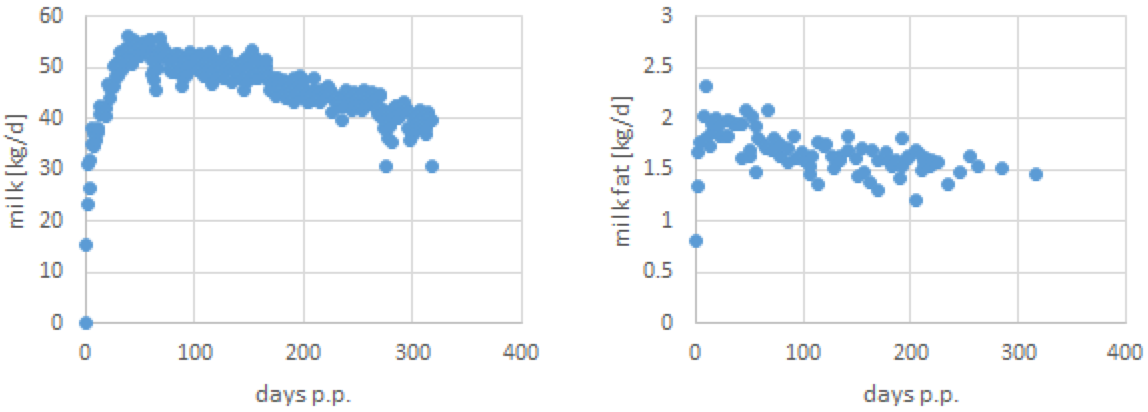

Figure S2. Milk (left) and milk fat (right) yield of control cow 3419.

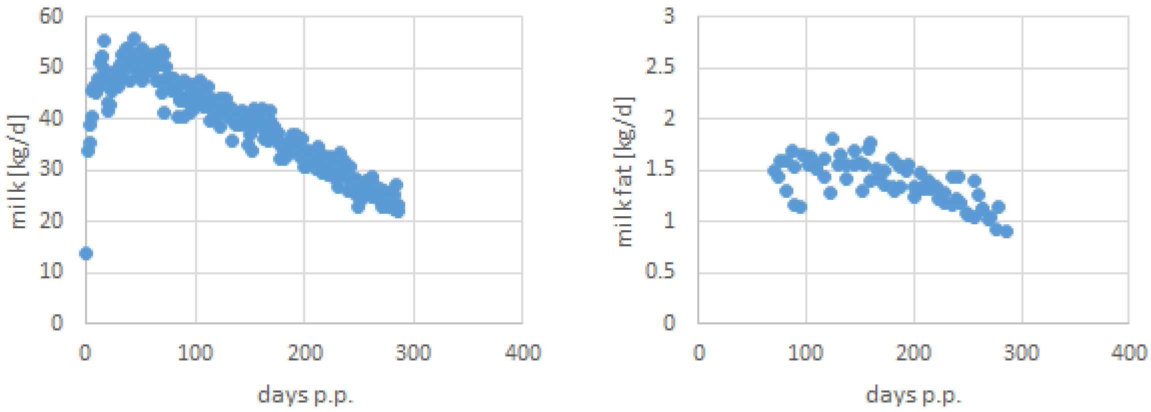

Figure S3. Milk (left) and milk fat (right) yield of control cow 3420.

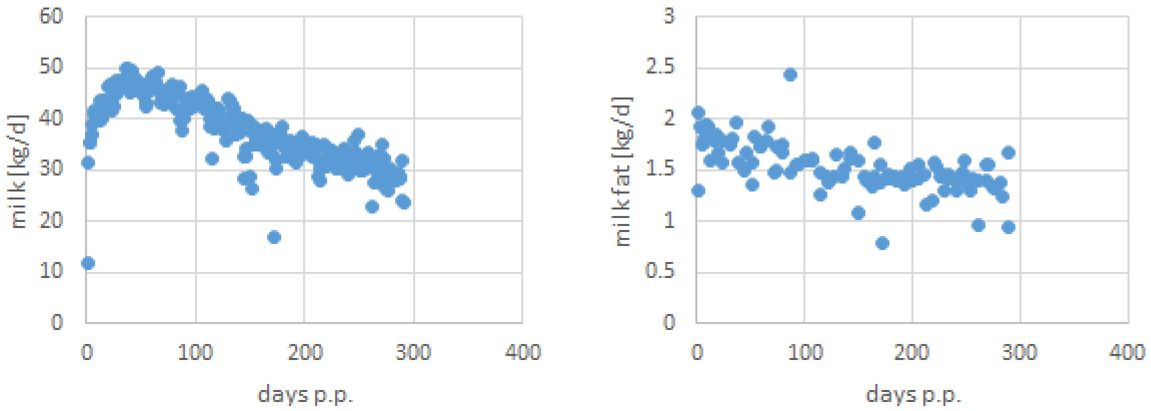

Figure S4. Milk (left) and milk fat (right) yield of control cow 3432.

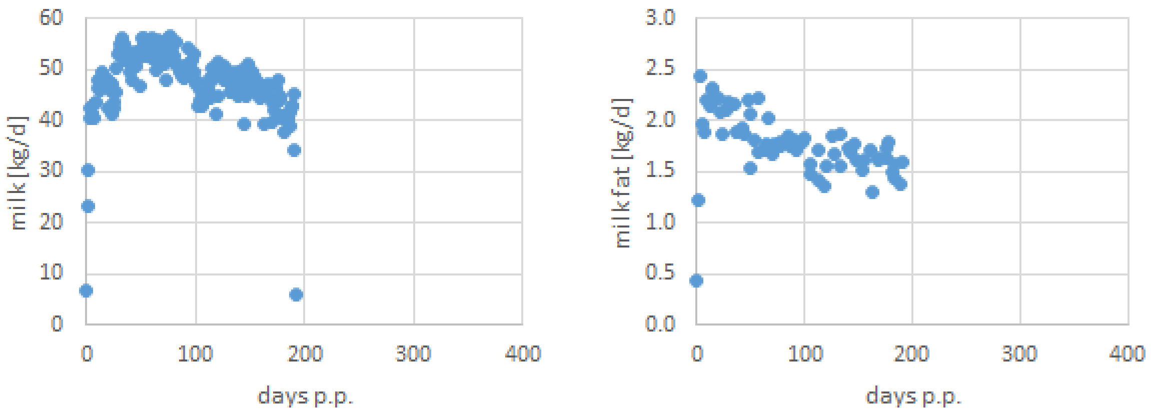

Figure S5. Milk (left) and milk fat (right) yield of control cow 3434.

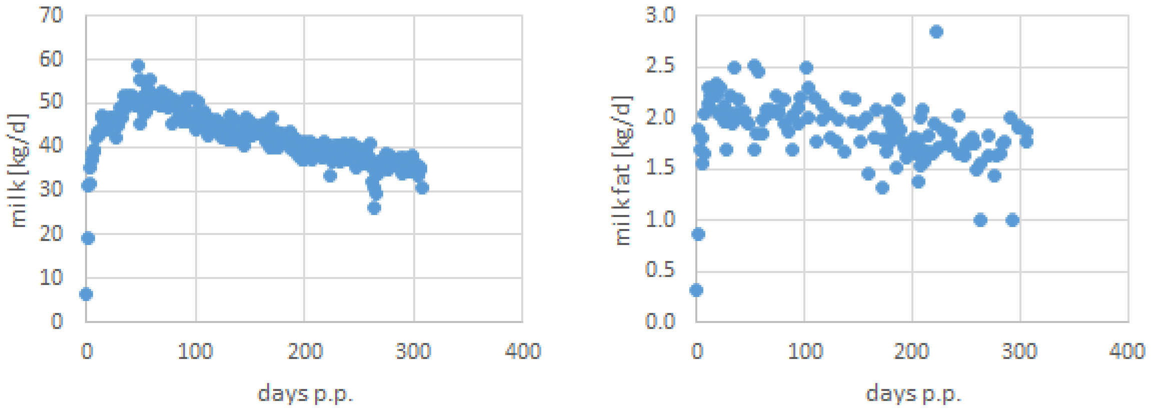

Figure S6. Milk (left) and milk fat (right) yield of experimental cow 3425.

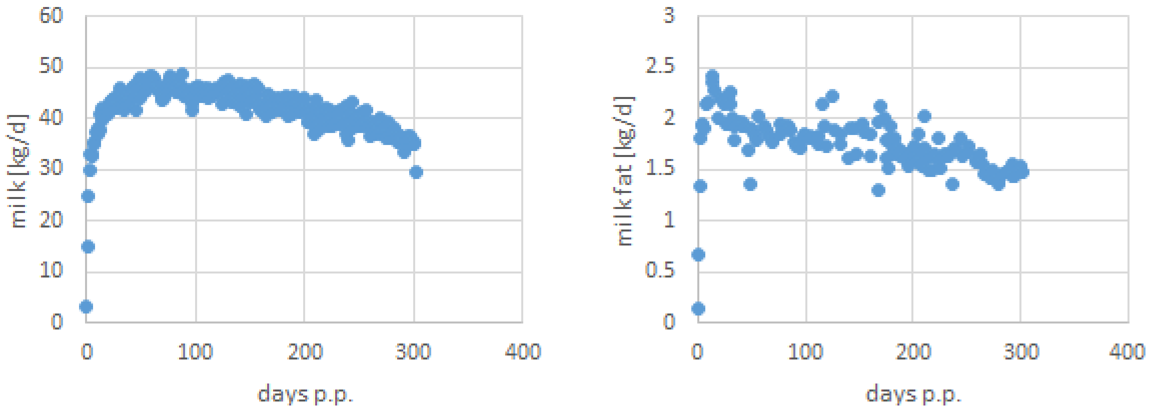

Figure S7. Milk (left) and milk fat (right) yield of experimental cow 3426.

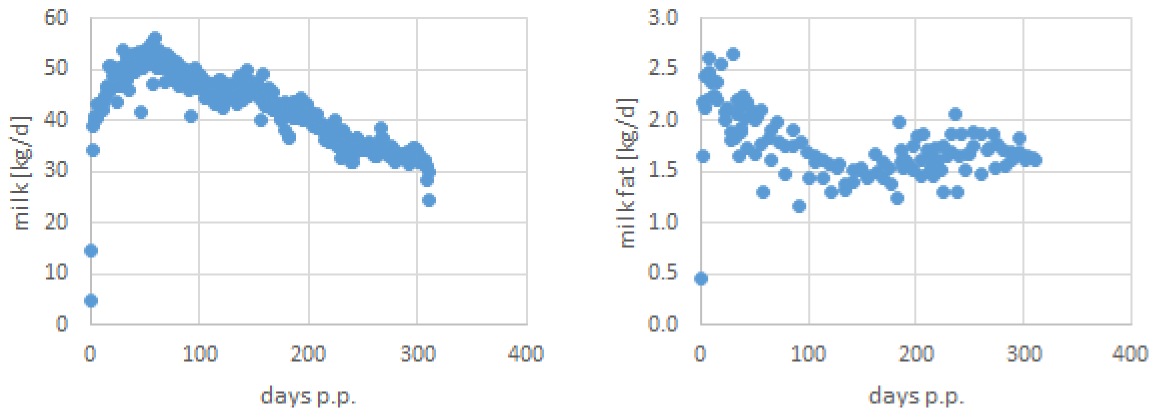

Figure S8. Milk (left) and milk fat (right) yield of experimental cow 3438.

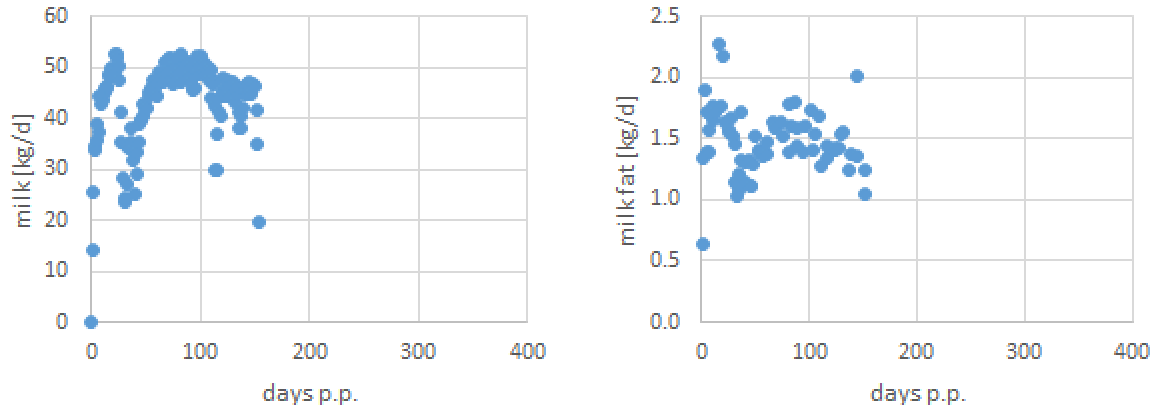

Figure S9. Milk (left) and milk fat (right) yield of experimental cow 3441.

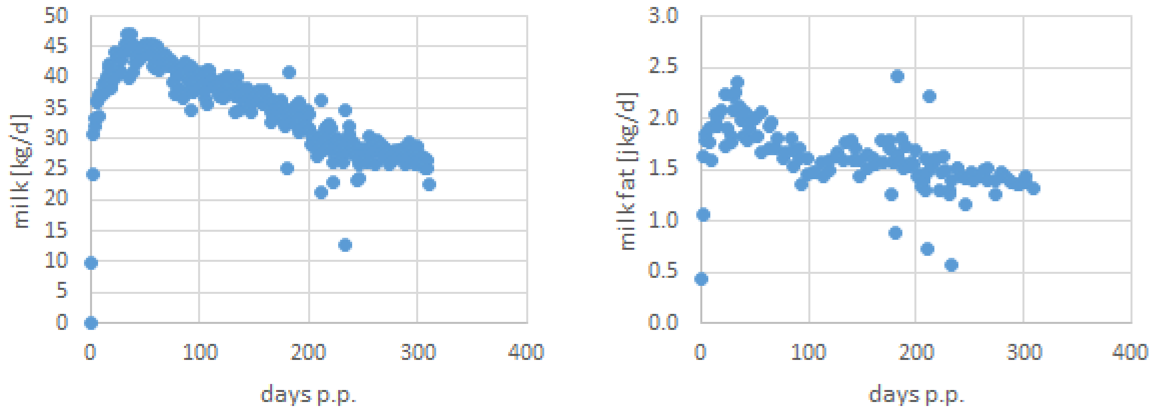

Figure S10. Milk (left) and milk fat (right) yield of experimental cow 3448.

Body weight

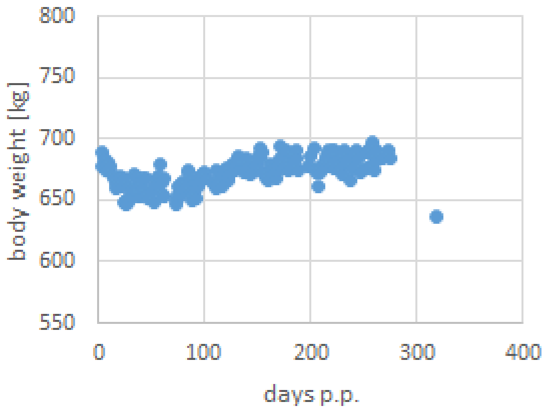

Figure S11. Body weight of control cow 3419.

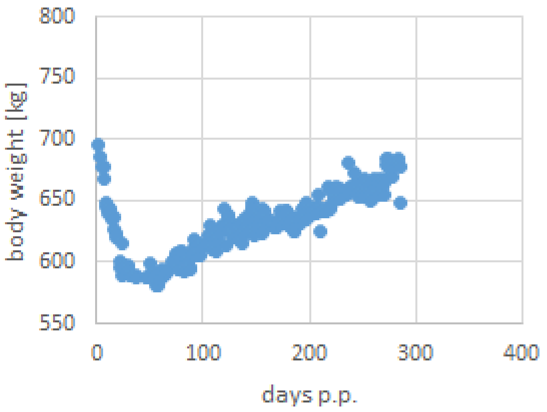

Figure S12. Body weight of control cow 3420.

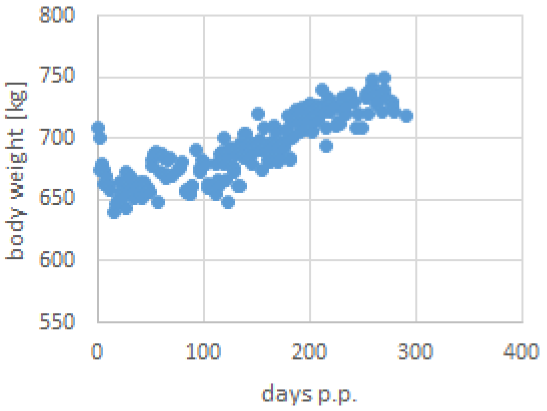

Figure S13. Body weight of control cow 3432.

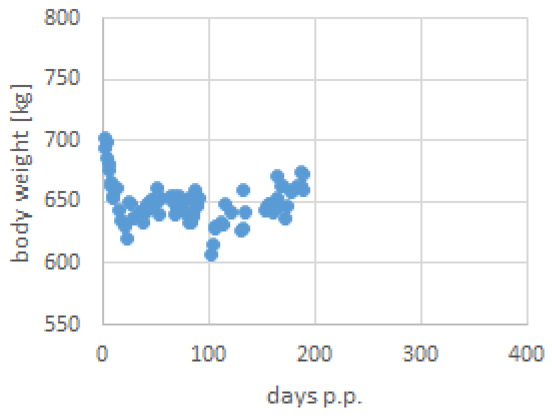

Figure S14. Body weight of control cow 3434.

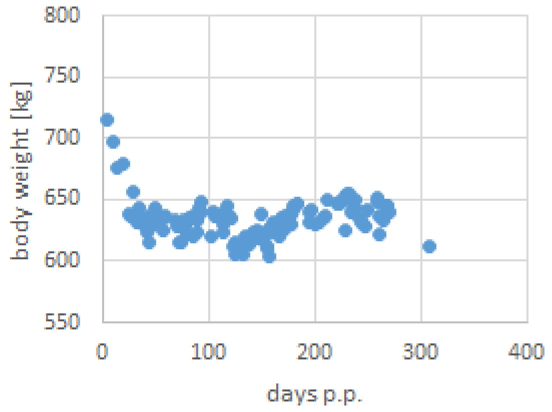

Figure S15. Body weight of experimental cow 3425.

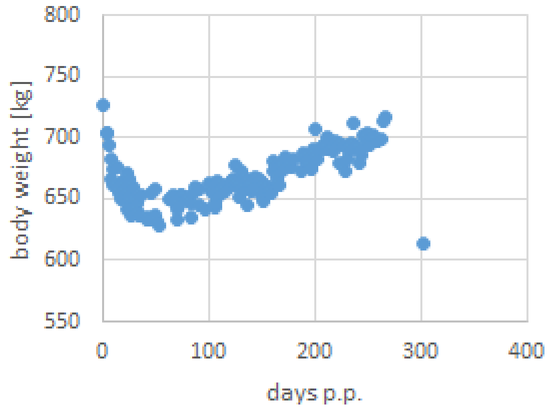

Figure S16. Body weight of experimental cow 3426.

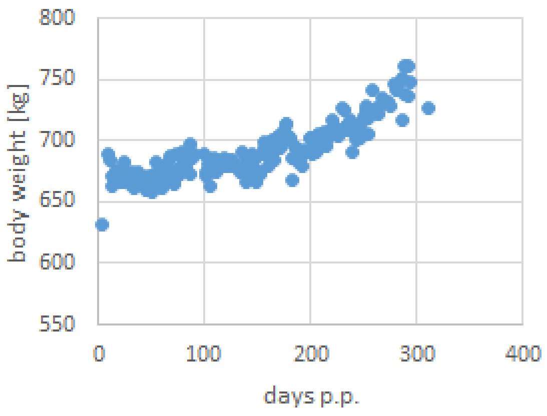

Figure S17. Body weight of experimental cow 3438.

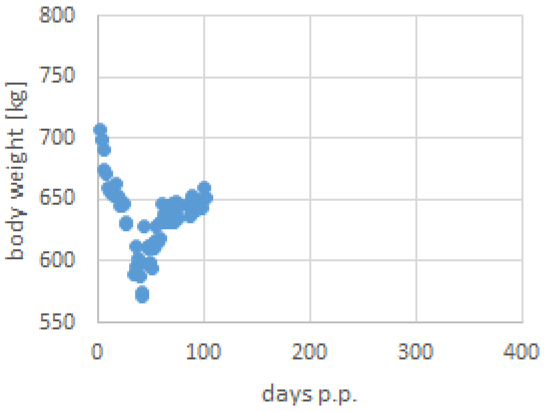

Figure S18. Body weight of experimental cow 3441.

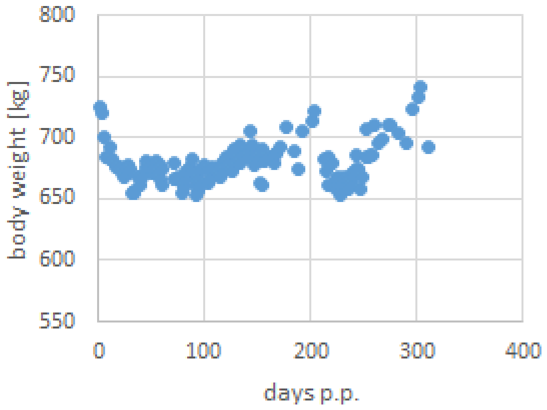

Figure S19. Body weight of experimental cow 3448.

Distribution of long chain fatty acids in milk

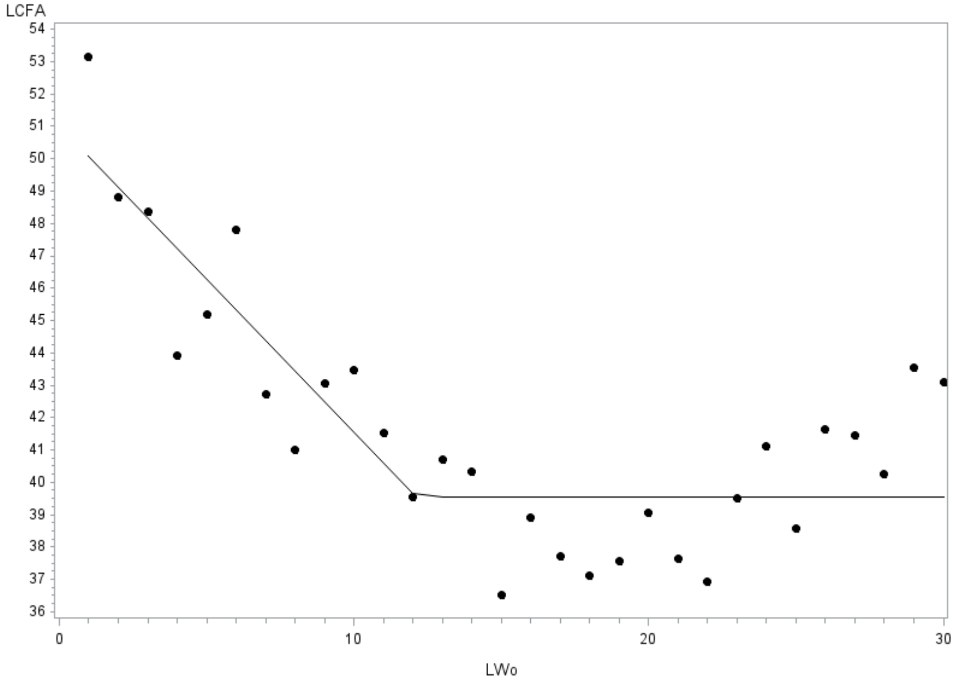

Figure S20. Percentage of long chain fatty acids (LCFA ≥ C18:0 in %, y-axis) in milk of control cow 3419 during its lactation weeks (LWo in weeks, x-axis).

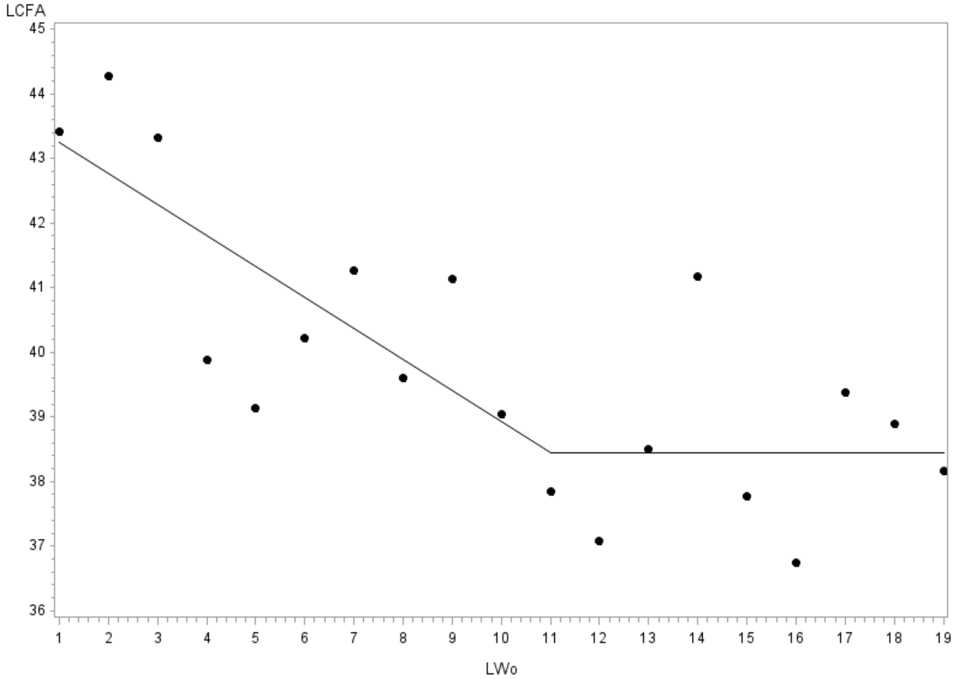

Figure S21. Percentage of long chain fatty acids (LCFA ≥ C18:0 in %, y-axis) in milk of control cow 3420 during its lactation weeks (LWo in weeks, x-axis).

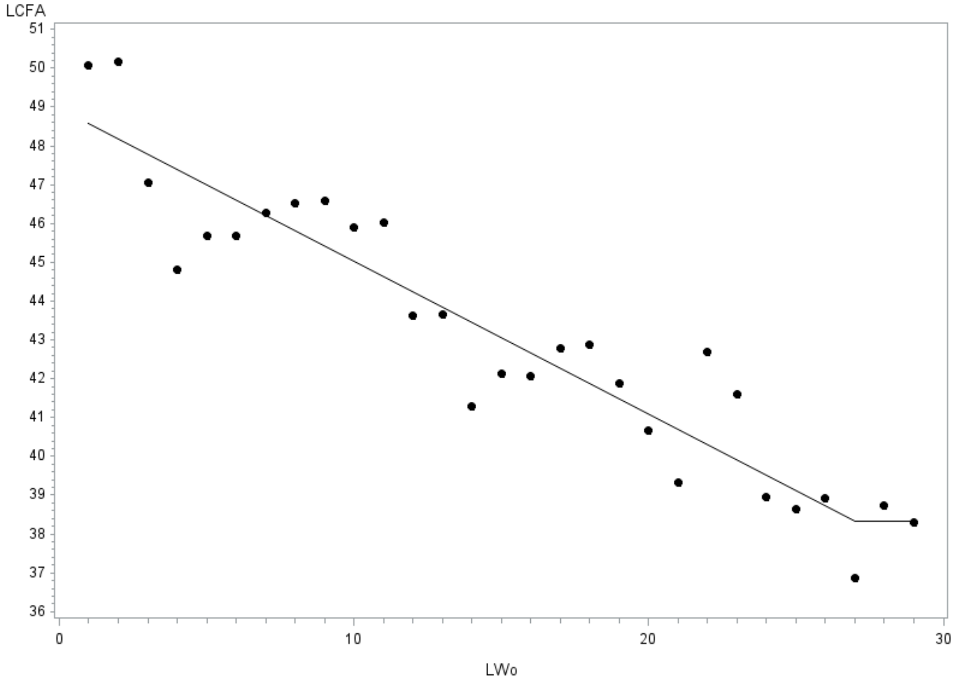

Figure S22. Percentage of long chain fatty acids (LCFA ≥ C18:0 in %, y-axis) in milk of control cow 3432 during its lactation weeks (LWo in weeks, x-axis).

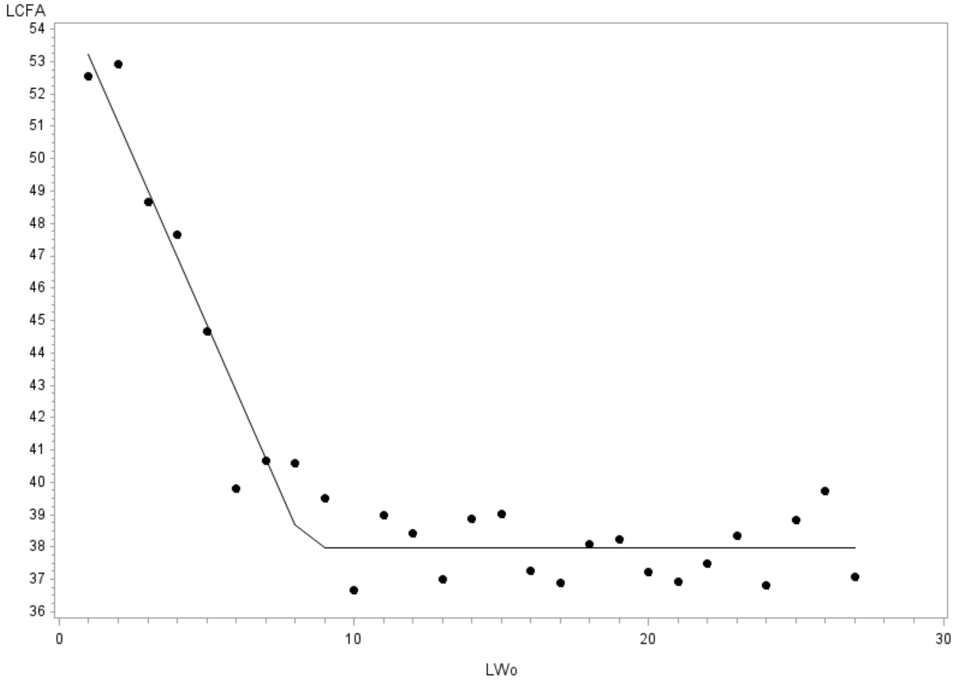

Figure S23. Percentage of long chain fatty acids (LCFA ≥ C18:0 in %, y-axis) in milk of control cow 3434 during its lactation weeks (LWo in weeks, x-axis).

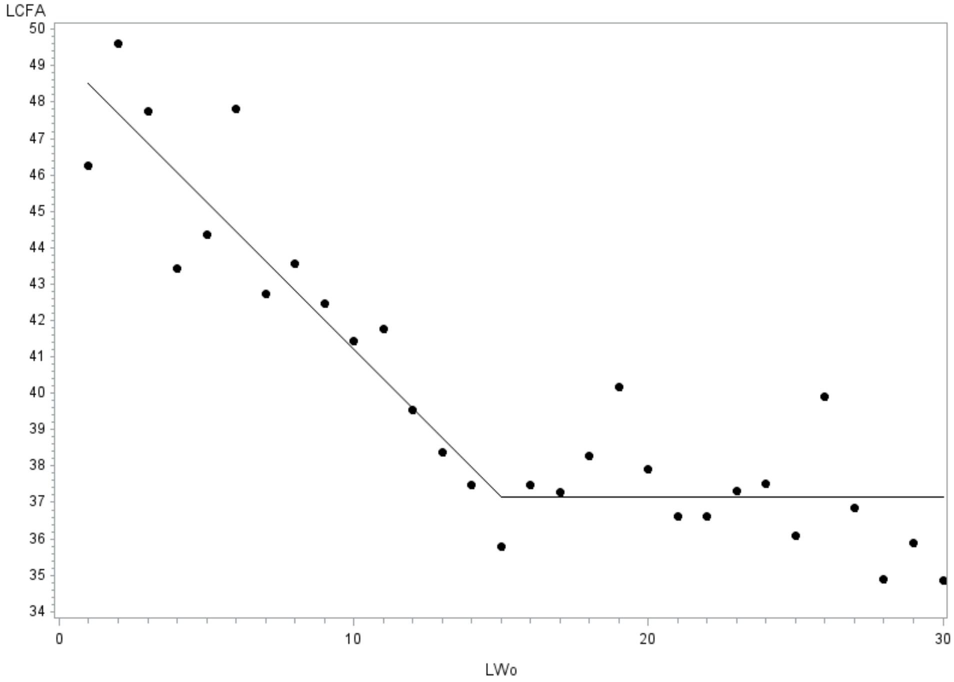

Figure S24. Percentage of long chain fatty acids (LCFA ≥ C18:0 in %, y-axis) in milk of experimental cow 3425 during its lactation weeks (LWo in weeks, x-axis).

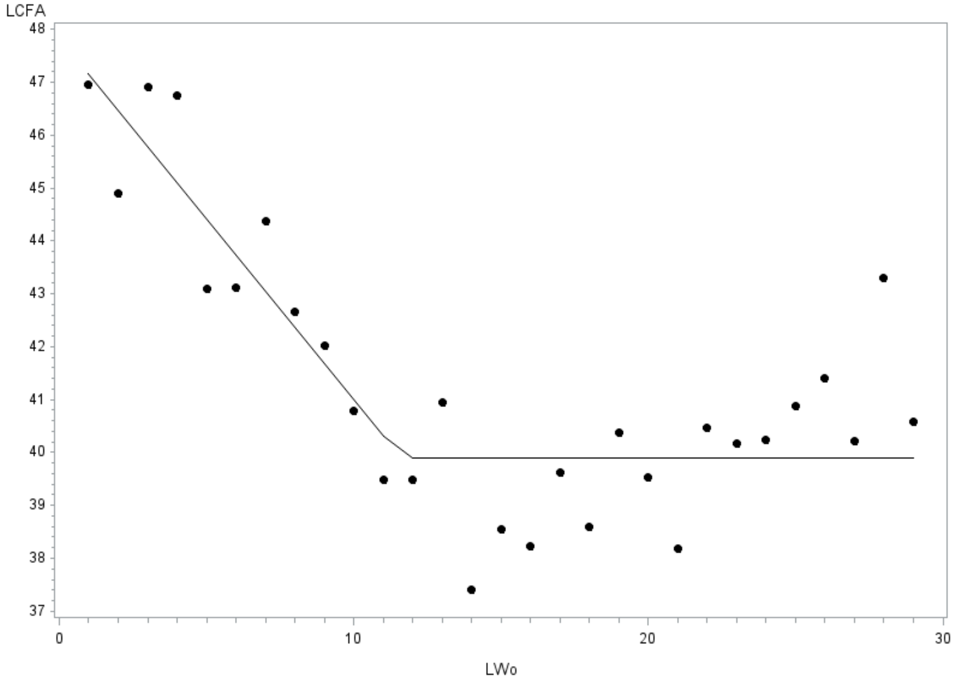

Figure S25. Percentage of long chain fatty acids (LCFA ≥ C18:0 in %, y-axis) in milk of experimental cow 3426 during its lactation weeks (LWo in weeks, x-axis).

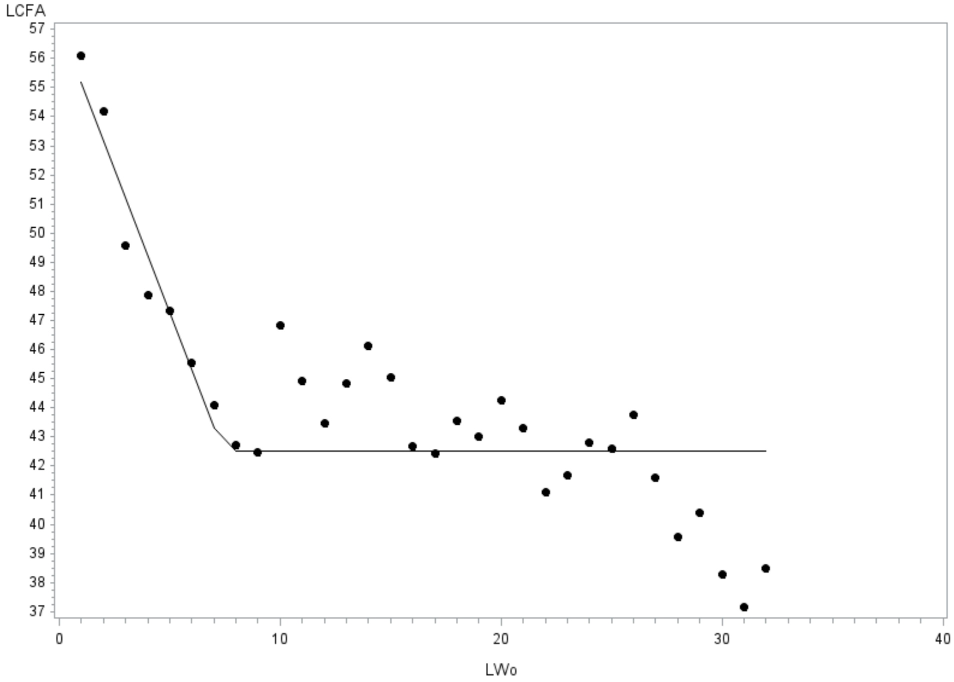

Figure S26. Percentage of long chain fatty acids (LCFA ≥ C18:0 in %, y-axis) in milk of experimental cow 3438 during its lactation weeks (LWo in weeks, x-axis).

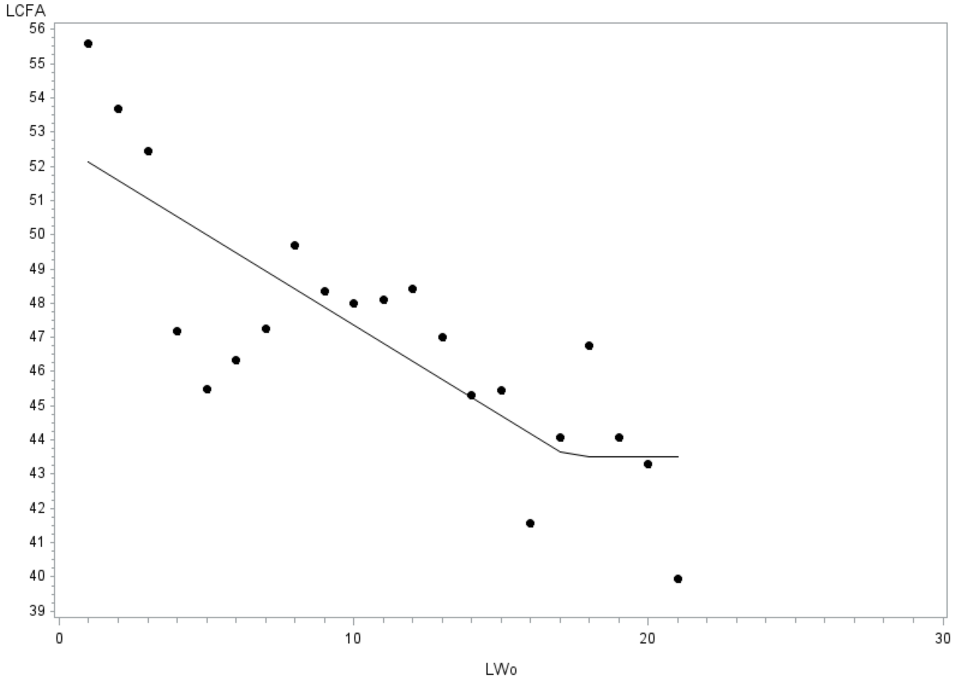

Figure S27. Percentage of long chain fatty acids (LCFA ≥ C18:0 in %, y-axis) in milk of experimental cow 3441 during its lactation weeks (LWo in weeks, x-axis).

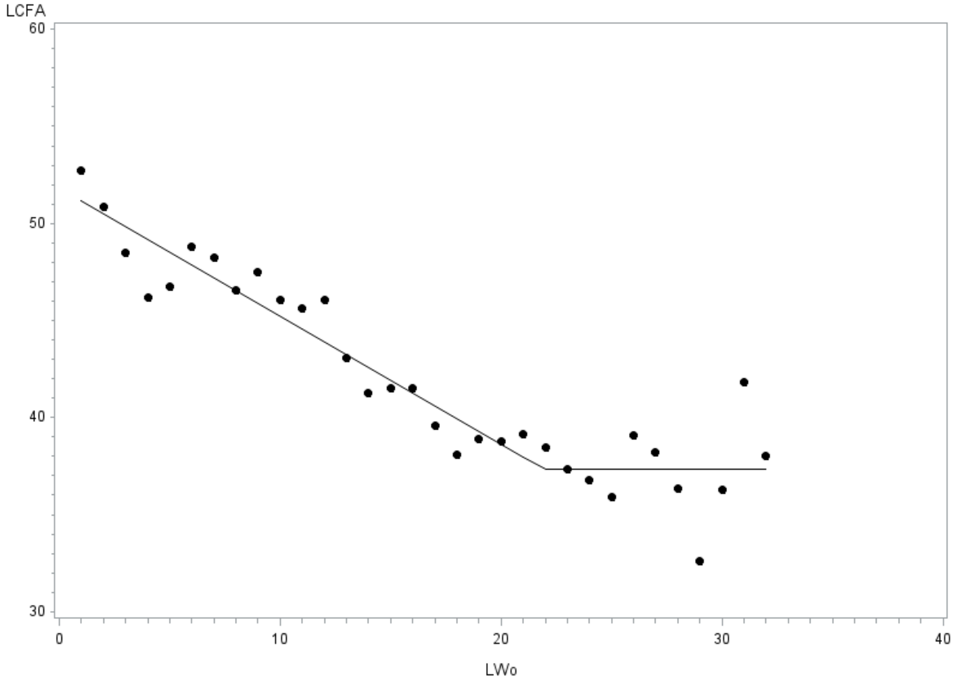

Figure S28. Percentage of long chain fatty acids (LCFA ≥ C18:0 in %, y-axis) in milk of experimental cow 3448 during its lactation weeks (LWo in weeks, x-axis).

Fecal crude fat

Table S4. Fecal crude fat values during the NEB dosing phase.

	cow ID number	fecal crude fat [%]
experimental	3425	5.25
	3426	2.23
	3438	2.38
	3441*	2.34
	3448	1.29
control	3419	2.40
	3420	no data
	3432	1.16
	3434*	0.95

*excluded from plotting in Figure S29.

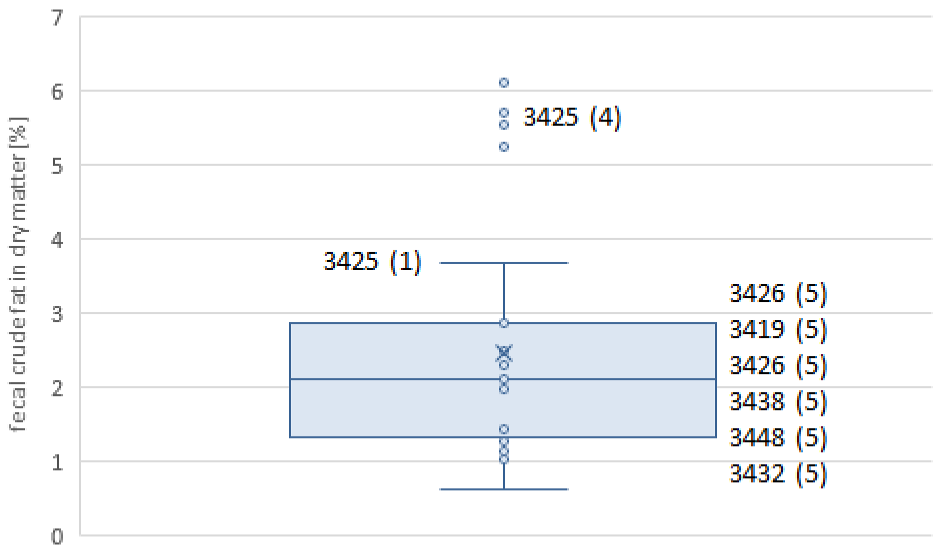

Figure S29. Distribution of fecal crude fat in samples of all cows except 3441 and 3434 during the NEB phase. Numbers in columns indicate the quantity of observations.

PCDD/F and PCB content in milk fat

Table S5. Mean PCDD/F and PCB content in milk fat of the control group between day 1 and 56 postpartum during the NEB phase.

cow ID number	3419	3420*	3432	3434**	mean for background correction
	[ng/kg]	[ng/kg]	[ng/kg]	[ng/kg]	[ng/kg]
2,3,7,8-TCDD	0.005		0.005	0.01	0.005
1,2,3,7,8-PeCDD	0.01		0.01	0.02	0.01
1,2,3,4,7,8-HxCDD	0.03		0.02	0.01	0.02
1,2,3,6,7,8-HxCDD	0.02		0.01	0.03	0.02
1,2,3,7,8,9-HxCDD	0.01		0.01	0.02	0.01
1,2,3,4,6,7,8-HpCDD	0.20		0.20	0.15	0.20
OCDD	1.75		1.27	1.55	1.51
2,3,7,8-TCDF	0.01		0.05	0.01	0.03
1,2,3,7,8-PeCDF	0.01		0.06	0.01	0.04
2,3,4,7,8-PeCDF	0.07		0.03	0.07	0.05
1,2,3,4,7,8-HxCDF	0.03		0.02	0.03	0.03
1,2,3,6,7,8-HxCDF	0.02		0.02	0.03	0.02
1,2,3,7,8,9-HxCDF	0.02		0.02	0.02	0.02
2,3,4,6,7,8-HxCDF	0.02		0.03	0.03	0.03
1,2,3,4,6,7,8-HpCDF	0.04		0.07	0.05	0.06
1,2,3,4,7,8,9-HpCDF	0.02		0.01	0.01	0.01
OCDF	0.23		0.20	0.21	0.22
PCB-28	1632.84		858.25	328.38	1245.54
PCB-52	65.09		43.28	50.74	54.19
PCB-101	74.83		103.14	53.39	88.98
PCB-138	460.64		472.62	449.50	466.63
PCB-153	622.75		626.12	581.10	624.43
PCB-180	256.09		229.79	206.42	242.94
PCB-77	2.15		1.71	2.16	1.93
PCB-81	0.27		0.25	0.22	0.26
PCB-105	39.05		32.18	28.25	35.61
PCB-114	2.85		1.68	2.82	2.27
PCB-118	195.12		173.22	161.55	184.17
PCB-123	0.06		0.06	0.06	0.06
PCB-126	2.61		2.86	2.79	2.73
PCB-156	30.11		24.98	26.24	27.55
PCB-157	3.26		2.93	3.66	3.10
PCB-167	18.34		20.96	19.37	19.65
PCB-169	0.17		0.28	0.20	0.22
PCB-189	3.59		3.40	3.55	3.49

* cow 3420 was included into the experiment after the NEB phase. ** Cow 3434 was excluded from mean background calculation due to health issues.

Table S6. Mean PCDD/F and PCB content in milk fat of the control group between day 179 and 234 postpartum during the PEB phase.

cow ID number	3419	3420	3432	3434*	mean for background correction
	[ng/kg]	[ng/kg]	[ng/kg]	[ng/kg]	[ng/kg]
2,3,7,8-TCDD	0.01	0.02	0.005		0.01
1,2,3,7,8-PeCDD	0.01	0.02	0.01		0.02
1,2,3,4,7,8-HxCDD	0.02	0.01	0.01		0.01
1,2,3,6,7,8-HxCDD	0.02	0.01	0.03		0.02
1,2,3,7,8,9-HxCDD	0.02	0.02	0.01		0.02
1,2,3,4,6,7,8-HpCDD	0.16	0.21	0.12		0.16
OCDD	1.69	1.23	0.63		1.18
2,3,7,8-TCDF	0.02	0.01	0.01		0.01
1,2,3,7,8-PeCDF	0.01	0.01	0.02		0.01
2,3,4,7,8-PeCDF	0.08	0.04	0.05		0.05
1,2,3,4,7,8-HxCDF	0.04	0.02	0.03		0.03
1,2,3,6,7,8-HxCDF	0.04	0.02	0.03		0.03
1,2,3,7,8,9-HxCDF	0.02	0.02	0.02		0.02
2,3,4,6,7,8-HxCDF	0.04	0.02	0.03		0.03
1,2,3,4,6,7,8-HpCDF	0.09	0.13	0.08		0.10
1,2,3,4,7,8,9-HpCDF	0.01	0.01	0.01		0.01
OCDF	0.10	0.43	0.16		0.23
PCB-28	2933.74	1361.59	1091.27		1795.53
PCB-52	91.46	52.43	46.94		63.61
PCB-101	225.74	57.99	60.69		114.81
PCB-138	676.13	397.69	351.12		474.98
PCB-153	836.14	450.03	447.21		577.79
PCB-180	320.44	206.93	176.97		234.78
PCB-77	1.65	1.34	1.21		1.40
PCB-81	0.19	0.24	0.11		0.18
PCB-105	35.51	25.27	22.54		27.77
PCB-114	1.91	1.77	1.45		1.71
PCB-118	196.77	133.67	127.07		152.50
PCB-123	0.06	0.06	0.06		0.06
PCB-126	1.72	2.05	1.88		1.89
PCB-156	32.90	21.86	20.69		25.15
PCB-157	2.97	2.19	2.23		2.47
PCB-167	21.38	14.14	12.90		16.14
PCB-169	0.18	0.19	0.14		0.17
PCB-189	4.32	2.79	2.84		3.32

*Cow 3434 was excluded from mean background calculation due to health issues.

Table S7. Mean PCDD/F and PCB content in milk fat of the experimental group between day 22 and 29 postpartum during the NEB phase.

cow ID number	3425 [ng/kg]	3426 [ng/kg]	3438 [ng/kg]	3441 [ng/kg]	3448 [ng/kg]
2,3,7,8-TCDD	2.02	2.71	3.15	3.43	3.65
1,2,3,7,8-PeCDD	2.79	4.13	4.55	5.64	5.95
1,2,3,4,7,8-HxCDD	3.47	4.45	5.40	6.16	6.88
1,2,3,6,7,8-HxCDD	2.26	2.93	3.87	4.32	4.50
1,2,3,7,8,9-HxCDD	2.37	2.92	3.89	4.50	4.97
1,2,3,4,6,7,8-HpCDD	8.90	7.40	11.50	12.35	14.94
OCDD	16.08	12.66	9.10	4.40	5.01
2,3,7,8-TCDF	4.73	4.99	4.83	4.80	6.27
1,2,3,7,8-PeCDF	9.08	11.19	10.85	11.10	14.28
2,3,4,7,8-PeCDF	2.62	3.70	4.27	4.67	5.21
1,2,3,4,7,8-HxCDF	3.56	4.79	5.85	6.44	7.20
1,2,3,6,7,8-HxCDF	2.31	2.58	3.44	3.45	4.16
1,2,3,7,8,9-HxCDF	2.01	2.15	2.20	2.62	2.91
2,3,4,6,7,8-HxCDF	3.13	3.74	4.70	5.54	6.15
1,2,3,4,6,7,8-HpCDF	3.42	3.26	4.73	5.25	6.04
1,2,3,4,7,8,9-HpCDF	2.90	2.48	3.42	3.78	4.35
OCDF	4.35	2.87	3.26	3.69	4.36
PCB-28	447.08	329.68	150.97	287.02	496.75
PCB-52	177.82	102.17	101.34	97.56	158.15
PCB-101	232.53	128.47	210.32	153.43	344.77
PCB-138	2342.66	2194.54	2625.39	2870.39	3225.05
PCB-153	1848.43	2005.05	2255.13	2604.91	2666.24
PCB-180	524.38	582.86	716.46	850.43	885.53
PCB-77	47.72	58.94	53.59	58.61	65.97
PCB-81	93.07	105.78	106.87	154.94	140.99
PCB-105	153.16	149.59	144.39	178.28	204.67
PCB-114	78.43	97.08	98.99	121.68	138.10
PCB-118	374.35	499.42	473.65	526.13	731.96
PCB-123	343.09	461.24	448.89	530.04	597.01
PCB-126	40.69	61.77	70.53	93.69	75.41
PCB-156	188.15	242.81	236.74	285.94	289.31
PCB-157	129.67	147.64	152.37	175.00	171.01
PCB-167	169.22	202.31	271.23	307.18	293.08
PCB-169	39.59	52.46	58.17	69.38	60.53
PCB-189	159.35	223.47	291.94	307.03	333.03

Table S8. Mean PCDD/F and PCB content in milk fat of the experimental group over the last week of the PEB dosing phase (around 200-207 days p.p.).

cow ID number	3425 [ng/kg]	3426 [ng/kg]	3438 [ng/kg]	3441 [ng/kg]	3448 [ng/kg]
2,3,7,8-TCDD	2.83	2.70	3.46		4.02
1,2,3,7,8-PeCDD	3.64	4.08	5.31		5.94
1,2,3,4,7,8-HxCDD	4.03	4.32	6.08		6.10
1,2,3,6,7,8-HxCDD	2.83	3.28	5.19		5.47
1,2,3,7,8,9-HxCDD	2.85	3.16	4.61		4.85
1,2,3,4,6,7,8-HpCDD	7.48	8.20	13.76		13.48
OCDD	8.88	8.64	11.63		4.38
2,3,7,8-TCDF	3.43	3.71	3.92		4.48
1,2,3,7,8-PeCDF	6.55	7.19	8.08		8.92
2,3,4,7,8-PeCDF	3.49	3.86	4.84		4.99
1,2,3,4,7,8-HxCDF	3.93	4.49	7.01		6.52
1,2,3,6,7,8-HxCDF	2.84	2.89	4.58		4.45
1,2,3,7,8,9-HxCDF	1.61	1.53	1.83		1.87
2,3,4,6,7,8-HxCDF	4.01	4.20	6.15		6.47
1,2,3,4,6,7,8-HpCDF	3.28	3.39	5.68		6.07
1,2,3,4,7,8,9-HpCDF	2.22	2.39	3.51		3.88
OCDF	1.88	2.31	3.19		2.97
PCB-28	585.99	301.72	814.69		1457.30
PCB-52	113.80	89.76	136.14		78.47
PCB-101	162.74	157.13	175.39		120.44
PCB-138	3231.87	2873.50	2602.31		3234.55
PCB-153	2472.65	2303.58	2170.86		2257.65
PCB-180	722.49	719.39	808.08		943.97
PCB-77	38.78	45.15	46.47		63.41
PCB-81	85.37	85.90	97.26		148.60
PCB-105	175.82	167.13	136.77		192.32
PCB-114	114.12	107.64	105.91		139.06
PCB-118	391.41	393.09	415.10		564.49
PCB-123	474.36	451.12	459.51		612.40
PCB-126	64.40	65.02	74.49		123.54
PCB-156	240.06	282.00	260.44		297.16
PCB-157	185.66	187.86	157.72		188.56
PCB-167	227.19	215.28	268.94		306.20
PCB-169	54.05	60.83	73.35		95.16
PCB-189	230.92	274.49	338.23		344.29

Transfer rates

Table S9. Mean PCDD/F transfer rates derived from the milk fat content during the last week of each dosing phase.

	**3425**		**3426**		**3438**		**3448**		**3441°**
	NEB	PEB	NEB	PEB	NEB	PEB	NEB	PEB	NEB
2,3,7,8-TCDD	23.3	26.3	33.6	24.9	33.1	28.6	31.5	28.3	27.5
1,2,3,7,8-PeCDD	20.6	24.3	32.3	27.0	30.5	29.8	31.0	25.2	29.2
1,2,3,4,7,8-HxCDD	17.0	16.3	23.2	17.3	23.7	21.0	23.7	17.2	20.8
1,2,3,6,7,8-HxCDD	14.9	16.7	20.7	19.3	23.6	23.3	21.0	20.8	20.3
1,2,3,7,8,9-HxCDD	10.6	10.5	13.9	11.6	15.4	14.8	15.8	12.6	13.7
1,2,3,4,6,7,8-HpCDD	3.9	2.7	3.4	2.9	4.5	3.9	4.5	3.4	3.7
OCDD*	0.7	0.2	0.5	0.2	0.4	0.3	0.1	0.1	0.1
2,3,7,8-TCDF	2.9	1.7	3.2	1.8	2.5	1.6	2.6	1.5	1.9
1,2,3,7,8-PeCDF	4.4	2.8	5.8	3.0	4.7	2.6	4.8	2.5	3.8
2,3,4,7,8-PeCDF	22.2	28.2	33.5	31.0	37.2	33.2	33.3	26.0	31.2
1,2,3,4,7,8-HxCDF	15.2	15.3	21.7	17.3	22.6	19.7	21.7	16.0	19.1
1,2,3,6,7,8-HxCDF	14.6	15.1	17.5	15.3	20.8	20.0	19.3	16.8	16.0
1,2,3,7,8,9-HxCDF	8.7	5.5	9.9	5.2	8.8	5.5	8.8	4.6	8.1
2,3,4,6,7,8-HxCDF	12.8	13.9	16.1	14.4	17.9	17.3	17.8	15.3	16.2
1,2,3,4,6,7,8-HpCDF	2.7	1.9	2.7	1.9	3.1	3.2	3.3	2.7	2.7
1,2,3,4,7,8,9-HpCDF	5.2	3.7	4.7	4.0	5.3	4.4	5.2	3.8	4.5
OCDF	0.5	0.2	0.3	0.3	0.4	0.3	0.4	0.2	0.3

*marks congeners that were also regularly present in method blanks. ° 3441 died before the PEB dosing regimen.

Table S10. Mean PCB transfer rates derived from the milk fat content during the last week of each dosing phase.

	**3425**		**3426**		**3438**		**3448**		**3441°**
	NEB	PEB	NEB	PEB	NEB	PEB	NEB	PEB	NEB
PCB-138*	34.9	40.3	34.3	34.8	34.2	27.6	33.7	27.5	26.0
PCB-153*	30.3	41.5	36.5	37.4	35.7	30.7	35.5	23.8	30.5
PCB-180*	26.8	31.2	35.0	30.7	35.3	34.4	37.2	33.6	31.2
PCB-77*	1.7	1.2	2.3	1.4	1.6	1.1	1.7	1.4	1.4
PCB-81	15.3	11.7	18.6	11.7	15.2	11.6	18.8	16.8	18.3
PCB-105*	30.8	32.8	31.7	30.7	24.6	19.5	30.0	23.7	22.5
PCB-144	30.3	38.4	40.4	35.8	33.8	31.4	39.3	32.3	30.6
PCB-118*	24.5	29.7	44.2	29.6	34.3	27.2	61.6	37.9	34.1
PCB-123	27.6	36.8	39.7	34.7	33.2	27.4	37.9	31.6	29.8
PCB-126	23.2	32.3	38.7	32.3	35.9	29.4	29.9	42.8	33.1
PCB-156*	28.0	34.4	40.4	40.7	32.2	30.5	35.1	29.7	30.6
PCB-157*	29.0	35.7	35.6	35.7	29.3	24.5	25.5	23.1	23.1
PCB-167*	31.9	36.8	41.2	34.3	41.3	36.0	41.2	35.8	38.5
PCB-169	28.7	35.3	40.7	39.3	36.2	38.1	31.4	41.9	31.8
PCB-189*	24.3	31.1	36.8	36.6	34.8	35.6	37.9	32.2	30.9

* marks congeners that were also present in method blanks. ° 3441 died before the PEB dosing regimen.

Congener Profiles

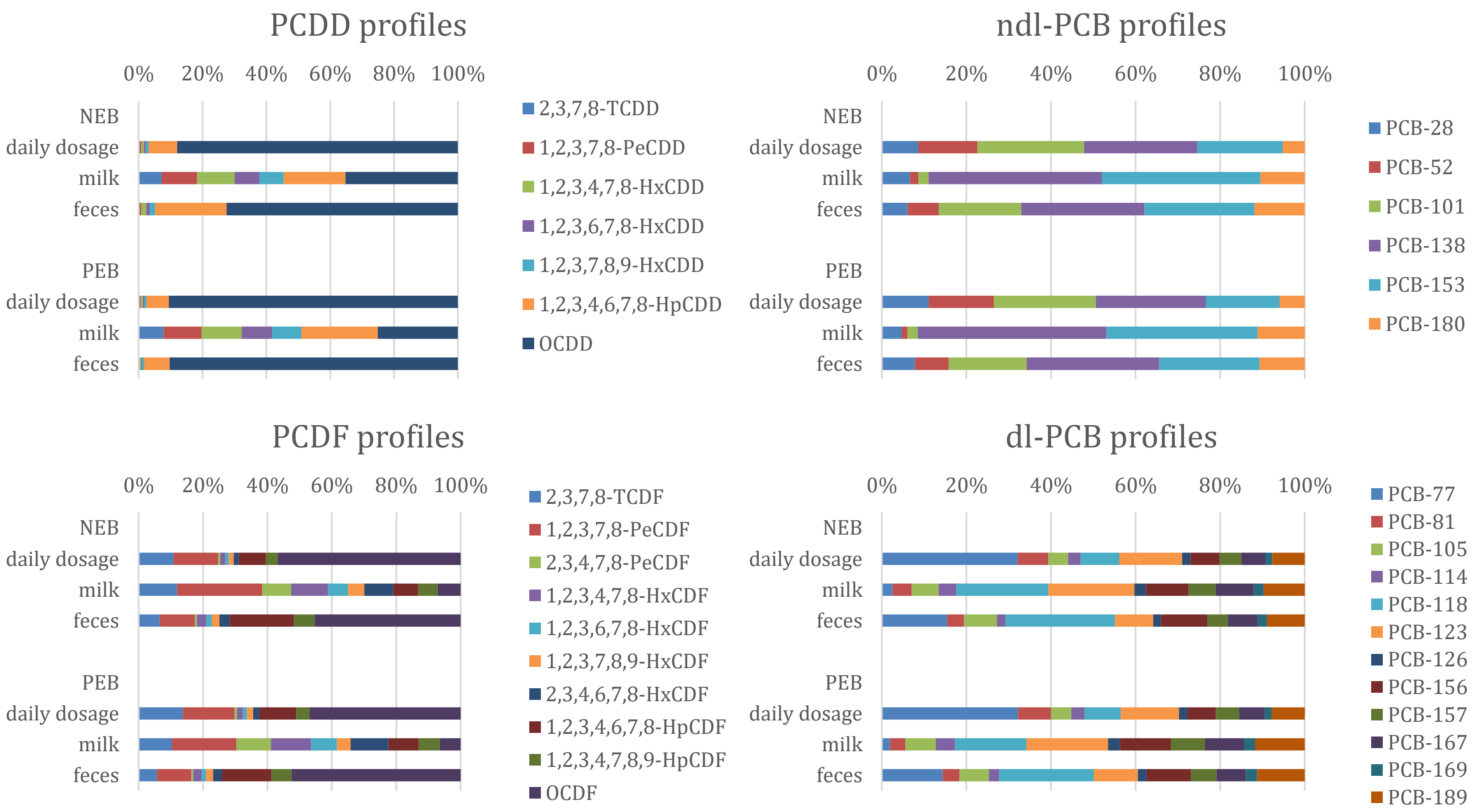

Figure S30. PCDD/F- and PCB-profiles for experimental cow 3426 at the end of each exposure phase (NEB and PEB).

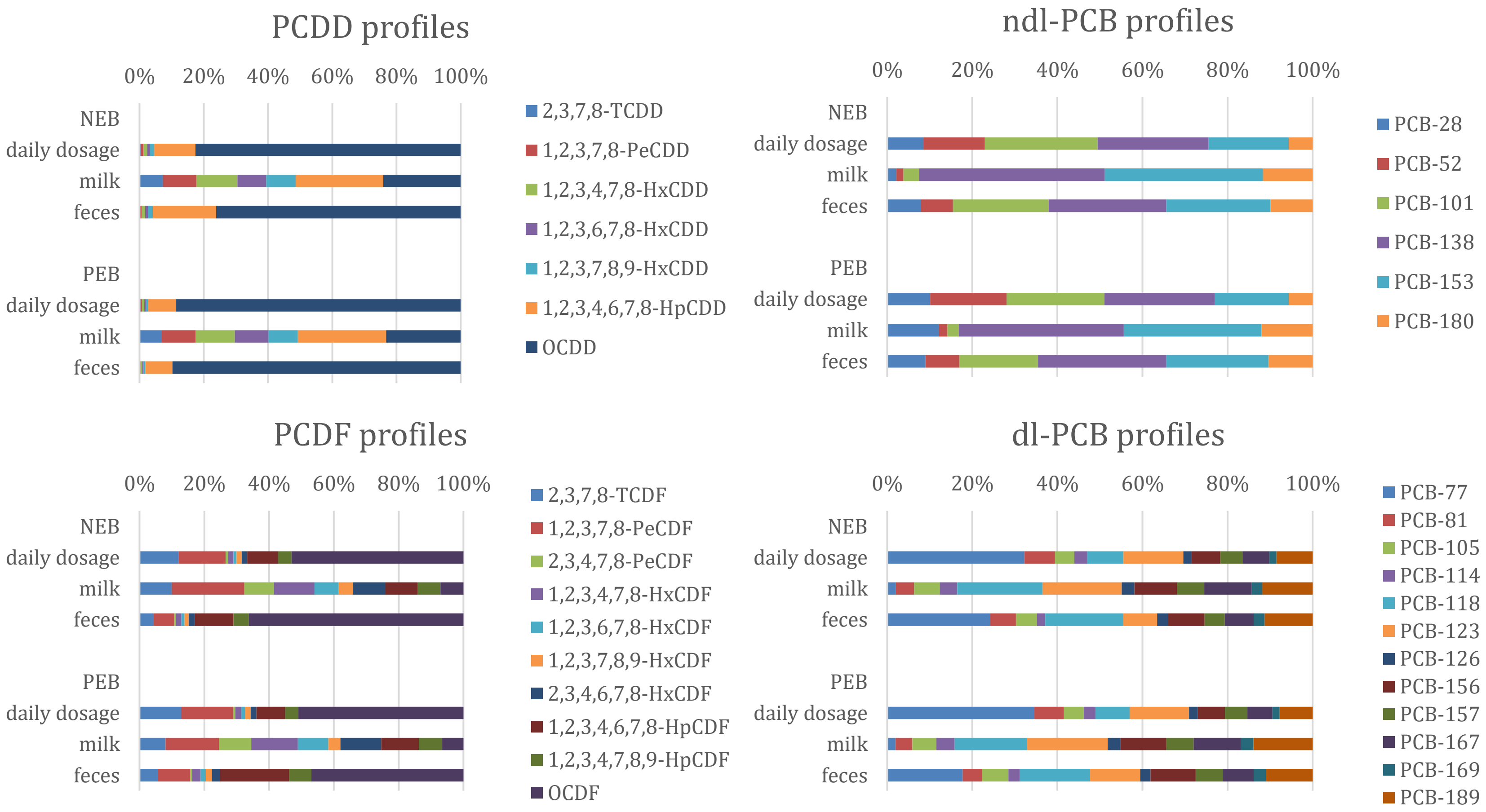

Figure S31. PCDD/F- and PCB-profiles for experimental cow 3438 at the end of each exposure phase (NEB and PEB).

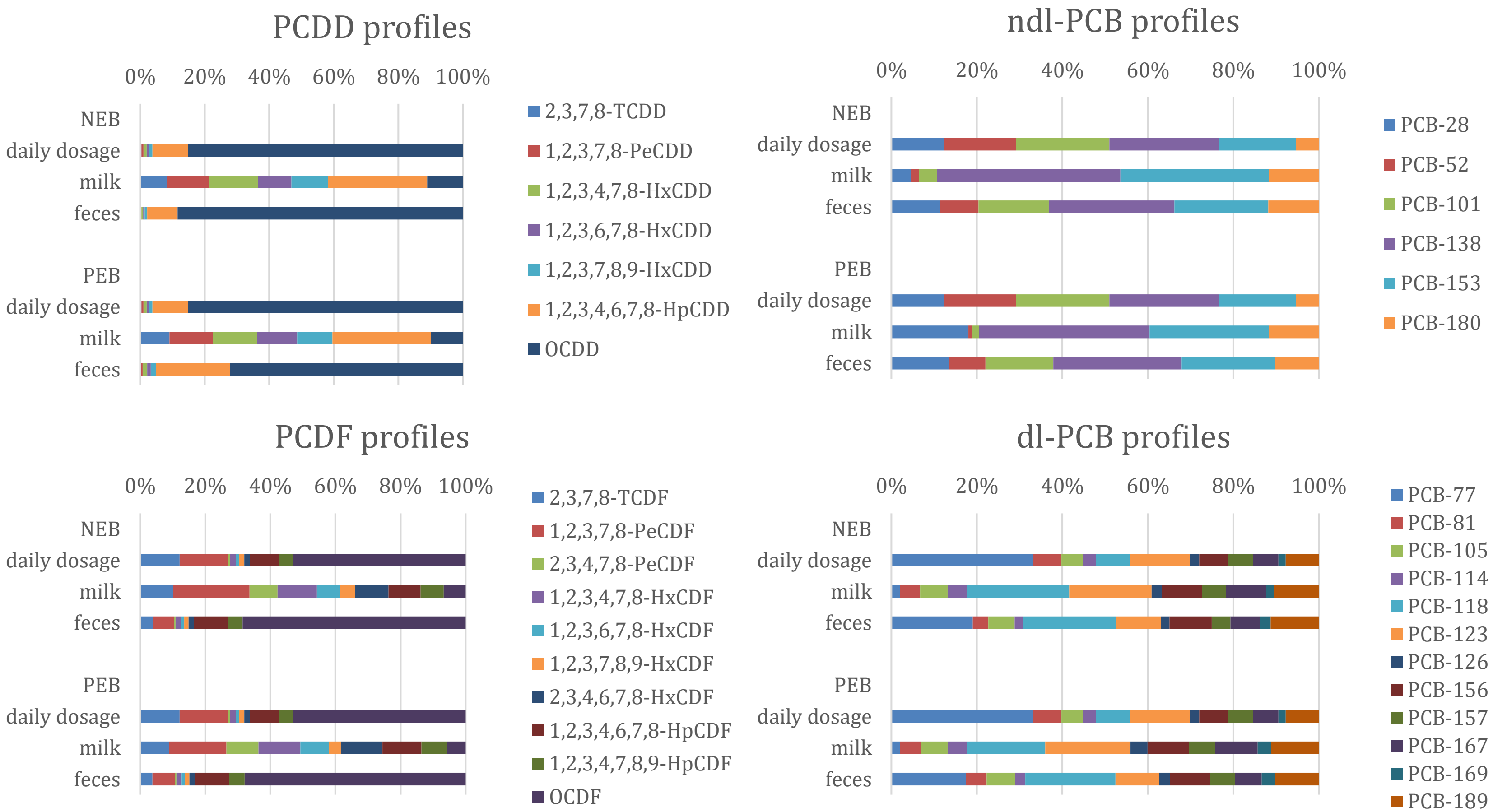

Figure S32. PCDD/F- and PCB-profiles for experimental cow 3448 at the end of each exposure phase (NEB and PEB).
